# Nitric Oxide in Biomaterial-Based Therapies for Coronary Heart Disease: Mechanistic Insights, Current Advances, and Translational Prospects

**DOI:** 10.34133/bmr.0267

**Published:** 2025-10-09

**Authors:** Jinpeng Sun, Zhiwen Wang, Yang Sun, Jiahui Zhang, Fangyuan Zhang, Junran Tong, Ran Gao, Xiaopeng Guo, Di Sun, Yumiao Wei

**Affiliations:** ^1^ Department of Cardiology, Union Hospital, Tongji Medical College, Huazhong University of Science and Technology, Wuhan 430022, China.; ^2^ Hubei Key Laboratory of Biological Targeted Therapy, Union Hospital, Tongji Medical College, Huazhong University of Science and Technology, Wuhan 430022, China.; ^3^ Hubei Engineering Research Center for Immunological Diagnosis and Therapy of Cardiovascular Diseases, Union Hospital, Tongji Medical College, Huazhong University of Science and Technology, Wuhan 430022, China.; ^4^ Department of Nephrology, Union Hospital, Tongji Medical College, Huazhong University of Science and Technology, Wuhan 430022, China.; ^5^ Department of Medical Records Management and Statistics, Union Hospital, Tongji Medical College, Huazhong University of Science and Technology, Wuhan 430022, China.; ^6^ School of Education, University of Nottingham, Nottingham, UK.; ^7^ Department of Radiology, Union Hospital, Tongji Medical College, Huazhong University of Science and Technology, Wuhan 430022, China.; ^8^ Department of Plastic Surgery, Union Hospital, Tongji Medical College, Huazhong University of Science and Technology, Wuhan 430022, China.

## Abstract

Coronary heart disease (CHD) remains a leading etiology of cardiovascular mortality globally. Endothelial dysfunction is now well-documented as the incipient pathological event in coronary atherosclerosis, with endogenous nitric oxide (NO) and nitric oxide synthase playing pivotal roles in regulating endothelial homeostasis via diverse signaling cascades. Over the past several decades, the pleiotropic functions of NO in cardiovascular physiology and pathophysiology have sparked substantial research interest in leveraging exogenous NO delivery strategies for atherosclerotic interventions. Beyond conventional NO-based pharmacotherapies, notable advancements have been achieved in the development of NO-releasing platforms and donor systems capable of spatiotemporally controlled and sustained NO delivery to target vascular tissues. This comprehensive review synthesizes current understanding of (a) the dual roles of endogenous NO in maintaining cardiovascular health and mediating pathological processes, (b) the enzymatic regulation of NO biosynthesis and its downstream signaling networks, and (c) the emerging translational potential of NO-based biomaterials in atherosclerotic management. Particular emphasis is placed on evaluating novel NO-donor systems and bioengineered constructs that exhibit therapeutic efficacy in preclinical models. Collectively, this analysis underscores the critical importance of NO-based biomaterials in advancing precision medicine approaches for CHDs, with implications for both diagnostic innovation and therapeutic optimization.

## Introduction

Cardiovascular disease (CVD) continues to be the leading cause of mortality worldwide [1]. Coronary heart disease (CHD) is defined as a pathological condition where atherosclerotic lesions in the coronary arteries lead to luminal stenosis or occlusion, culminating in myocardial ischemia, hypoxia, and necrosis. With an estimated global prevalence of 240 million cases of ischemic heart disease [2], surgical interventions—including percutaneous coronary intervention (PCI) and coronary artery bypass grafting (CABG)—remain the cornerstone of mortality reduction strategies. Notwithstanding substantial advancements in surgical techniques and device development, restenosis remains an inevitable complication in CHD patients during long-term follow-up, primarily attributed to blood–material interface interactions and neointimal hyperplasia (NIH) [3].

In the late 20th century, nitric oxide (NO) was identified as the endothelium-derived relaxing factor (EDRF) responsible for vascular smooth muscle relaxation. Subsequent studies have established that endogenous NO functions as a pleiotropic signaling molecule orchestrating diverse cellular processes, including vasodilation, immune modulation, neurotransmission, apoptosis, reproductive physiology, and gene transcriptional regulation [4]. Within endothelial cells, NO synthesized by nitric oxide synthase (NOS) serves as a critical regulator of vascular homeostasis, playing dual roles in mediating both protective and pathological responses in cardiovascular systems. Mechanistically, this gaseous signaling molecule exerts its cytoprotective effects through multiple pathways: (a) inhibiting vascular smooth muscle cell (VSMC) proliferation and migration, (b) attenuating platelet aggregation and thrombus formation, and (c) suppressing leukocyte adhesion and activation, thereby mitigating inflammatory responses [5,6]. Emerging evidence highlights the therapeutic potential of NO-donor molecules and bioengineered systems capable of delivering NO in a spatiotemporally controlled manner for atherosclerotic interventions. The development of sustained-release NO-releasing biomaterials represents a promising strategy to address the unmet clinical needs in restenosis prevention and atherosclerotic plaque stabilization, by recapitulating the multifaceted protective functions of endogenous NO at targeted vascular sites.

**Fig. 1.**
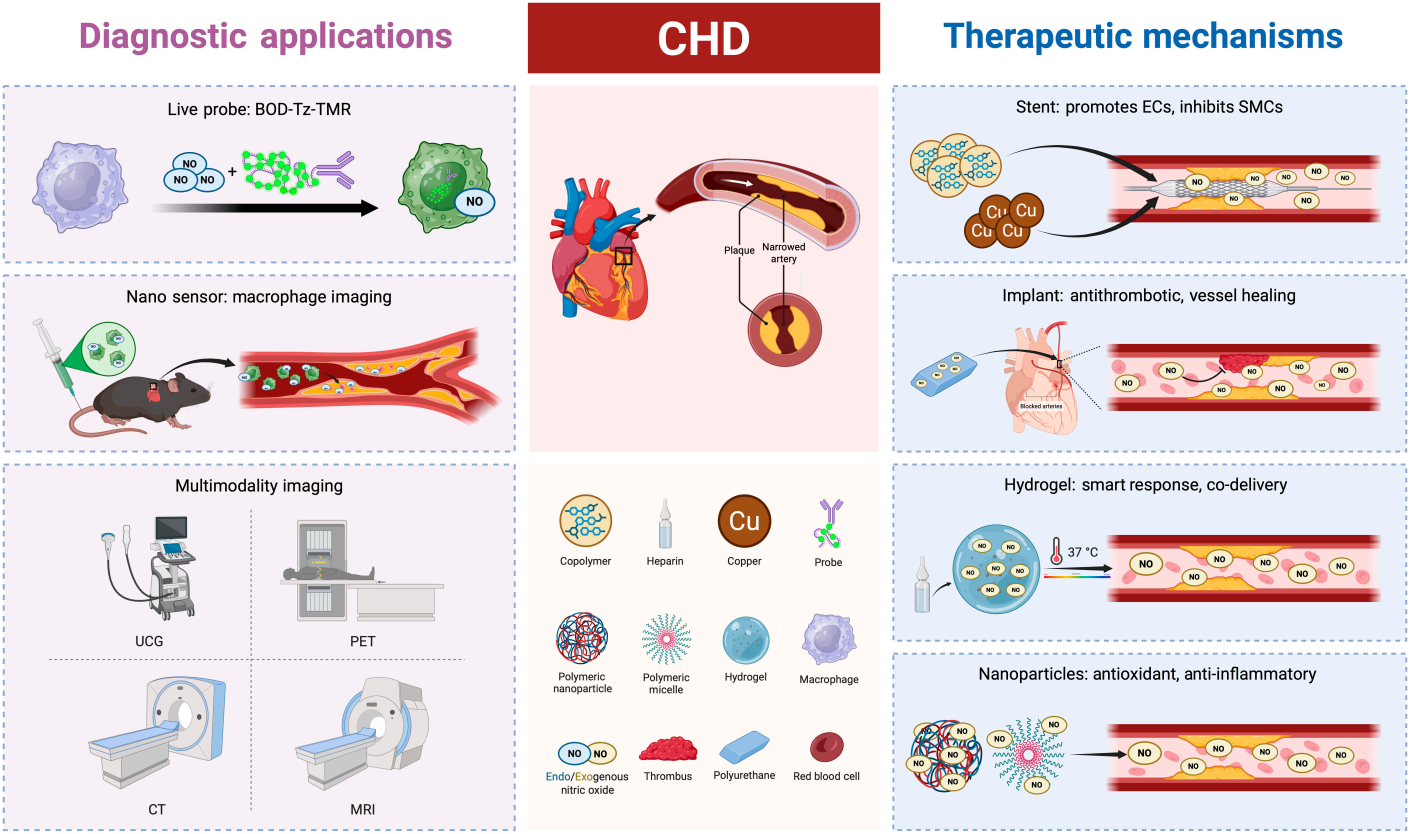
Diagnostic and therapeutic strategies of CHD based on NO.

This review provides an in-depth analysis of the physiological and pathological roles of NO in the cardiovascular system, with a focus on its relationship with endogenous NO, NOS, and atherosclerosis. We also examine NO detection techniques based on diverse methodologies and explore the medical applications of exogenous NO-based biomaterials in atherosclerosis treatment (Fig. 1). Notably, this review highlights the pivotal role of NO-based stents and vascular grafts in preventing restenosis and other adverse outcomes following coronary artery surgery. By emphasizing the latest advancements in NO-based biomaterials, particularly nanomaterials, we aim to provide new insights into the development of NO release systems for improved therapeutic outcomes in cardiovascular medicine.

## Endogenous NO

### Discovery of endogenous NO

NO, a diffusible gaseous signaling molecule, was initially characterized as the EDRF in cardiovascular systems. Landmark studies by Katsuki et al. [7] revealed nitrite-mediated activation of guanylate cyclase (cGMP) leading to vasorelaxation, hypothesizing NO as the bioactive mediator. Furchgott and Zawadzki [8] further demonstrated that acetylcholine-induced vasodilation requires intact endothelial cells to release NO, which diffuses to adjacent VSMCs causing relaxation. NO research is complicated by its nanoscale molecular size (1 nm), ultrashort intravascular half-life (2 ms), variable extracellular stability (0.09 to >2 s dependent on oxygen tension), high diffusivity, and rapid reactivity with biomolecules [9]. Combined with its ultralow physiological concentrations (≤100 pM to ~5 nM) and heterogeneous cellular distribution, these properties pose substantial challenges for NO detection [10,11]. Consequently, elucidating NO’s role in cardiovascular pathophysiology remains technically challenging.

### Function of endogenous NO

Over the past several decades, researchers have intensively investigated NO in terms of its synthesis, distribution, biological properties, and functional roles, yielding valuable insights into its pivotal role within the cardiovascular system [12,13]. NO is synthesized from L-arginine (L-Arg) and oxygen via the catalytic action of NOS, which exists in 3 distinct isoforms: neuronal NOS (nNOS), endothelial NOS (eNOS), and inducible NOS (iNOS) [14].

#### Physiological function of NO in blood vessels

**Fig. 2.**
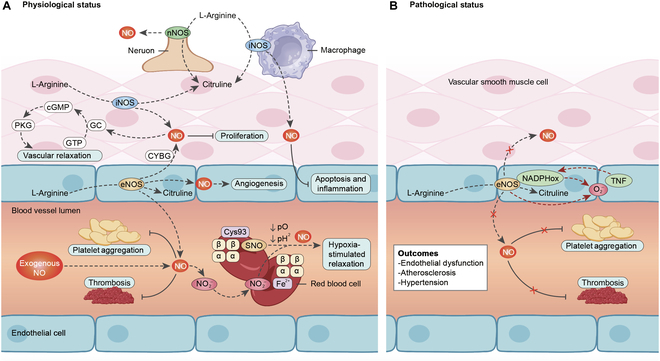
Physiological (A) and pathological (B) function of NO inside blood vessels.

Under physiological conditions, eNOS-derived NO diffuses to VSMCs and activates the sGC–cGMP–PKG cascade, lowering intracellular Ca^2+^ by stimulating plasma membrane calcium ATPase and sarcoplasmic/endoplasmic reticulum calcium ATPase activities and coordinating ion-channel control [15,16]. PKG further limits Ca^2+^ entry by promoting membrane hyperpolarization and reducing L-type Ca^2+^ channel activity [17–19], and it facilitates myosin light-chain dephosphorylation to support relaxation [20]. Paracrine and mechanical cues additionally modulate NO generation and downstream signaling in vascular smooth muscle [21–24] (Fig. 2A). Although NO is freely diffusible, its vasodilatory and antiplatelet actions occur predominantly within near-wall microdomains, effectively restricting functional effects to the peri-endothelial region [25,26]. Consistent with these mechanisms, experimental and clinical evidence further shows that NO preserves endothelial barrier integrity, sustains shear-dependent microvascular perfusion, and reinforces antithrombotic and anti-inflammatory homeostasis [27–30].

#### Pathological function of NO in blood vessels

Under pathological conditions, cardiovascular risk factors disrupt eNOS coupling and diminish NO bioavailability [31]. Oxidative depletion of tetrahydrobiopterin (BH_4_) and related redox imbalance favor superoxide production and peroxynitrite (ONOO^−^) formation, aggravating endothelial dysfunction [32–35]. Loss of NO signaling promotes leukocyte adhesion, smooth-muscle proliferation, platelet aggregation, and pro-thrombotic responses, thereby accelerating vascular disease progression [36–38]. These alterations integrate with metabolic and inflammatory stressors to amplify vascular injury [39]. Beyond local synthesis, disturbances in NO production, concentration, and diffusion are shaped by red-blood-cell scavenging/transport dynamics [40,41] and by microbiome-dependent nitrate–nitrite–NO pathways that influence vascular homeostasis [42]. Collectively, both insufficient and dysregulated/excess NO perturb vascular equilibrium, underscoring the need to restore balanced NO signaling in disease [43,44] (Fig. 2B).

## NO and Atherosclerosis

### Atherosclerosis, endothelial dysfunction, and NO

Atherosclerosis is a chronic, progressive arteriopathy characterized by arterial wall sclerosis and luminal narrowing. Its pathogenesis involves endothelial dysfunction, lipoprotein oxidation, and macrophage-derived proinflammatory mediators, driving VSMC proliferation, lipid accumulation, and platelet aggregation within the intima [45,46]. Endothelial dysfunction arises from an imbalance between vasoprotective factors (e.g., NO and endothelium-dependent hyperpolarization) and increased oxidative stress and vasoconstrictors [47,48]. Substantial evidence indicates that NO-dependent endothelial dysfunction is the earliest event in atherogenesis [49–51], even preceding hypertension in both human and animal models [52]. The seminal study by Ludmer et al. [53] further confirmed the central role of NO in human atherosclerosis using coronary angiography.

### NOS and atherosclerosis

All 3 NOS isoforms are implicated in the regulation of vascular homeostasis and atherosclerotic pathogenesis (Table 1). Genetic ablation studies in murine models have demonstrated that nNOS and eNOS exert protective effects against atherosclerotic lesion formation, whereas iNOS promotes pro-atherogenic processes.

**Table 1.** Functional roles of NOS isoforms and their impact on atherosclerosis

Isoform	Expression pattern	Physiological role	Dysregulation/Mechanism	Net effect in atherosclerosis
eNOS (NOS3)	Endothelium (caveolae, myoendothelial junctions)	Vasodilation; anti-platelet; anti-inflammatory; inhibits VSMC proliferation/migration	Uncoupling (BH_4_ ↓, ADMA ↑, S-glutathionylation) → ↓NO, ↑O_2_^−^/ONOO^−^; oxidative stress and endothelial dysfunction	**Protective** eNOS^−^/^−^ (ApoE^−^/^−^) mice → accelerated lesions; overexpression under hyperlipidemia/oxidative stress aggravates disease via uncoupling [61]
iNOS (NOS2)	Macrophages, VSMCs, ECs (inducible)	Ca^2+^-independent, high-output NO; immune defense	Sustained overproduction → ONOO^−^ formation; nitroxidative stress; feed-forward with NOX/COX2 → vascular injury	**Pathogenic (high-output)** iNOS^−^/^−^ (ApoE^−^/^−^) mice → reduced plaque burden and lipid peroxidation; inhibitory effect most evident in advanced lesions [77]
nNOS (NOS1)	Perivascular nerves, mural cells, cardiomyocytes	Maintains basal flow; neurovascular coupling; regulates vascular tone and autonomic control	Dysregulated signaling → autonomic imbalance; excessive ROS → endothelial dysfunction and remodeling	**Predominantly protective (context-dependent)** nNOS^−^/^−^ (ApoE^−^/^−^) mice → larger plaques and higher mortality; pharmacological inhibition lowers basal coronary flow in humans [80]

BH_4_, tetrahydrobiopterin; ADMA, asymmetric dimethylarginine; ONOO^−^, peroxynitrite; VSMC, vascular smooth muscle cell; EC, endothelial cell

#### eNOS and atherosclerosis

eNOS, a Ca^2+^/calmodulin-dependent enzyme, is central to endothelial protection and anti-atherogenic defense [54–57]. Constitutively expressed in endothelial cells, eNOS is activated by shear stress or agonists (e.g., bradykinin and acetylcholine), and its NO diffuses to VSMCs to stimulate cGMP-dependent vasodilation. Endothelial NO also limits platelet adhesion/aggregation and thrombus formation, while restraining low-density lipoprotein (LDL) oxidation, VSMC proliferation, and leukocyte adhesion [56,58].

eNOS uncoupling represents a key pathogenic mechanism in atherosclerosis. Cardiovascular risk factors provoke oxidative stress and endothelial dysfunction, with impaired acetylcholine/bradykinin vasodilatory responses and a shift toward eNOS uncoupling [55,59]. In apolipoprotein E-knockout (ApoE^−/−^) mice, reduced endothelial NO bioavailability reflects both superoxide-mediated NO scavenging and eNOS uncoupling [60]. Genetic eNOS ablation exacerbates lesion development, whereas paradoxically, eNOS overexpression can induce relative BH_4_ deficiency, promote uncoupling, and accelerate disease [61]. Enhancing BH_4_ biosynthesis via GTP-cyclohydrolase I overexpression reduces plaque burden, increases vascular BH_4_, and lowers superoxide derived from uncoupled eNOS [61]. Notably, coupled and uncoupled eNOS can coexist within the same cell; uncoupling-driven oxidative stress does not negate the vasculoprotective effects of coupled eNOS-derived NO [62].

Recent work adds transcriptional/post-transcriptional regulation to this framework: endothelial OASL1 deficiency accelerates atherosclerosis by aggravating endothelial dysfunction and down-regulating NOS3 (eNOS-encoding gene) via miR-584-dependent mRNA destabilization; inhibition of miR-584 reverses this phenotype [63].

eNOS uncoupling, where the enzyme produces superoxide instead of NO, is a pivotal driver of endothelial dysfunction. This shift impairs vasodilation, increases oxidative stress, and promotes vascular inflammation and remodeling, thereby contributing to hypertension and atherogenesis. Key mechanisms include depletion of the cofactor BH_4_, accumulation of endogenous inhibitor asymmetrical dimethylarginine (ADMA), and redox-sensitive post-translational modifications [64,65].

Paradoxically, eNOS overexpression can worsen oxidative stress when the redox balance is disrupted. Excess enzyme activity may outstrip the supply of BH_4_ or L-Arg, leading to uncoupling and superoxide production instead of NO. This creates a self-perpetuating cycle of oxidative damage. Thus, the vascular outcome depends not only on eNOS levels but also critically on its coupling status [65].

Endothelial BH_4_ deficiency directly triggers eNOS uncoupling, impairing coronary vasodilation and aggravating ischemia–reperfusion injury. Nur77 overexpression increases eNOS and GCH1, lowers ROS, and improves acetylcholine-mediated relaxation, whereas Nur77 deletion worsens dysfunction. DDAH1 overexpression reduces ADMA, preserving endothelium-dependent vasodilation, limiting remodeling, and attenuating inflammation in Ang II models [66–68]. Collectively, these findings highlight that NO insufficiency and eNOS uncoupling are central drivers of vascular injury, underscoring the therapeutic importance of restoring NO homeostasis.

#### iNOS and atherosclerosis

iNOS is inducible in platelets, VSMCs, and macrophages, modulating cardiovascular NO signaling [69]. Its NO output is concentration-dependent—lower levels may be protective, whereas sustained high production contributes to vascular injury and atherogenesis [70]. Although iNOS is absent under physiological conditions, oxidative and inflammatory cues induce its expression, causing persistent, high NO that associates with vasoconstriction and endothelium-dependent vasodilatory dysfunction [71]. A central downstream mechanism is ONOO^−^, which mediates tissue injury across atherosclerosis, myocardial infarction, and chronic heart failure [72]. ONOO^−^ formed via Nox2/COX2-derived superoxide further induces iNOS expression, creating a feed-forward loop [73,74]. In diseased human coronary arteries, iNOS colocalizes with COX2 and nitrotyrosine in macrophage-rich regions [75]; iNOS is also present in human coronary atherosclerotic plaques and closely associated with ONOO^−^ formation [75,76]. Consistently, ApoE/iNOS double-knockout mice show reduced atherosclerosis not through altered lipoprotein profiles but via lower circulating lipid peroxides, implicating iNOS-driven nitroxidative stress in lesion progression [77].

#### nNOS and atherosclerosis

nNOS is constitutively expressed in perivascular neural plexuses and vascular mural cells [78]. Vascular nNOS exerts local regulatory effects independently of central nervous system-derived nNOS signaling [56]. In ApoE^−/−^ mice, nNOS deletion increases atherosclerotic plaque formation in the aortic root and descending thoracic aorta, and atherogenic diet further elevates mortality in nNOS/ApoE double knockouts [79,80]. In carotid ligation models, nNOS-knockout mice exhibit accelerated vascular remodeling compared with wild-type controls [81]. Pharmacological nNOS inhibition with S-methyl-L-thiocitrulline selectively reduces basal coronary blood flow without altering substance P-induced vasodilation [82].

Beyond NO, nNOS-related ROS (e.g., H_2_O_2_ and superoxide) act as signaling intermediates in nonneuronal compartments [83]. Current work is defining the nNOS–H_2_O_2_ axis in human internal mammary arteries and saphenous veins used for CABG, with coronary-derived H₂O₂ implicated in pro-atherogenic remodeling and vein-graft dysfunction [84,85]. In humans, nNOS polymorphisms (rs2293050 T and rs2139733 A) have been associated with large-vessel ischemic stroke risk [86]. Together, these data support a predominantly vasculoprotective role for nNOS in early atherogenesis, modulated by context-dependent ROS/nitrosative pathways.

Overall, NO signaling in the vasculature is organized around a few design-critical principles. Coupled eNOS activity ensures physiological NO production, while uncoupling under oxidative stress generates superoxide and peroxynitrite, aggravating vascular dysfunction. Canonical sGC–cGMP–PKG signaling coexists with cGMP-independent pathways such as protein S-nitrosylation, which modulate inflammation, mitochondrial function, and cell survival. The nitrate–nitrite–NO axis maintains vasodilation under hypoxic or acidic conditions, complementing oxygen-dependent eNOS activity. Recent evidence highlights RBC–endothelium crosstalk and the role of mobile NO-ferroheme species in distributing vasodilatory bioactivity throughout the vascular wall. Finally, subcellular localization of eNOS—within caveolae, endosomes, and myoendothelial junctions—creates spatially restricted signaling domains critical for specificity. Together, these advances argue that biomaterials should (a) preserve eNOS coupling, (b) deliver NO within physiological flux ranges, and (c) exploit nitrite/S-nitrosothiols (RSNOs) reservoirs while avoiding peroxynitrite-mediated toxicity [87].

## Detection of NO and Diagnosis of Atherosclerosis

NO mediates pleiotropic vascular effects, underscoring the critical need for novel NO biosensors in cardiovascular bioimaging. These sensors show promise as disease biomarkers for subclinical screening, objective progression assessment, and treatment adherence monitoring. Two translational strategies have emerged: real-time in situ tissue imaging and systemic blood NO quantification. In situ NO imaging may prognosticate atherosclerotic plaque progression and identify high-risk patients. Systemic NO levels could predict complications including fibroproliferative remodeling, plaque erosion, and vulnerable plaques associated with acute cardiovascular events. Collectively, quantifying NO bioavailability in blood and plaques offers clinical utility for disease monitoring, therapeutic evaluation, and adherence assurance in cardiovascular care.

However, direct detection of NO in vivo is particularly challenging due to its intrinsic physicochemical properties. As a highly diffusible free radical, NO rapidly permeates membranes and disperses from its generation site, limiting spatial resolution. Its ultrashort half-life (milliseconds to seconds) and rapid reactions with hemoglobin and reactive oxygen species (ROS) produce secondary derivatives such as peroxynitrite, complicating measurement specificity. Moreover, the physiological NO concentration is typically within the pico- to nanomolar range, often below the sensitivity threshold of conventional assays. These features collectively underscore the need for advanced nanosensors and probe-based technologies with high temporal and spatial precision.

### In situ tissue imaging of NO

Macrophages critically contribute to atherosclerotic plaque pathogenesis by forming cholesterol-rich foam cells in the necrotic core [88]. Upon activation, they induce iNOS expression, producing NO at concentrations up to ~1 μM during experimental validation [89]. Thus, quantitative mapping of NO in plaque macrophages or iNOS-active cells provides insight into microenvironments predisposing to erosion or rupture. In vitro, suspended macrophages are widely used to assess NO sensor performance [90,91]; RAW 264.7 cells have served as standard models, and 2-photon probes enable exogenous NO imaging in rodent tissues [92–95].

Recently, Lin et al. reported a “live-sensor” system: a tetrazine-based ratiometric probe introduced via metabolic labeling and bio-orthogonal chemistry. This platform selectively targeted atherosclerotic lesions as small as tens of micrometers (Fig. S1), and ratiometric signals correlated endogenous NO production with lesion severity, establishing a powerful approach for probing plaque microenvironments [96]. Unlike endothelial-surface imaging, macrophages’ phagocytic capacity facilitates nanoparticle (NP)-mediated delivery of NO sensors. Bifunctional metallic NPs combining contrast imaging with NO sensing are promising for vulnerable-plaque risk stratification. Future refinements include targeting monocyte subsets by cluster differentiation markers and engineering macrophage-specific nanosensors. Translational applications already extend to computed tomography, magnetic resonance imaging, and positron emission tomography, and analogous molecular reporters may enable in situ macrophage imaging across atherosclerosis and other CVDs [97,98].

Beyond macrophage-targeted approaches, several optical probes have expanded the scope of in situ NO imaging within vascular tissues. Boron-dipyrromethene (BODIPY)- and rhodamine-derived fluorescent sensors enable dynamic visualization of NO release in endothelial and inflammatory cells with subcellular resolution [99]. Near-infrared and 2-photon probes, such as silicon-rhodamine derivatives, provide deeper tissue penetration and reduced background autofluorescence, making them suitable for monitoring NO in atherosclerotic lesions [100]. More recently, bio-orthogonal and ratiometric strategies have been used to improve selectivity and quantification of endogenous NO at disease sites [96]. Collectively, these imaging modalities complement macrophage-specific probes and establish a versatile toolkit for studying endothelial dysfunction and plaque inflammation in situ.

### Blood NO quantification

Because of NO’s extremely short half-life, systemic evaluation usually relies on its stable metabolites, nitrite and nitrate (NOx), or on NO-derived adducts. Chemiluminescence assays remain the reference method for plasma NOx, though dietary intake and renal clearance can confound interpretation [101]. Electron paramagnetic resonance spectroscopy allows direct detection of hemoglobin–NO complexes in blood, providing a closer reflection of bioactive NO levels, but requires strict handling to avoid artifacts [102]. More recently, liquid chromatography–mass spectrometry (LC-MS)/MS) has enabled targeted quantification of circulating RSNOs and other NO-derived species with high specificity [103]. Clinically, altered blood NOx concentrations have been associated with endothelial dysfunction and increased cardiovascular risk in prospective cohorts [104]. Although blood-based assays cannot fully capture local vascular NO dynamics, they offer a practical and clinically accessible biomarker strategy that complements tissue-level imaging.

### Nano-biomaterials for NO detection

Nanomaterials provide multifunctional platforms for imaging, drug delivery, and therapy, and have been engineered to detect reactive oxygen and nitrogen species both in vitro and in vivo. Park et al. [105] reported a gold NP-based NO activator that uses NO as a dual marker for macrophage-specific targeting and ablation, with potential translation to intravascular NO imaging and cardiovascular applications. Diverse nanoplatforms—lipid-, metal-, and carbon-based—have been conjugated with fluorescent probes to achieve spatiotemporally resolved NO detection. By exploiting NP delivery, sensors can be directed to subcellular sites such as lysosomes. Surface functionalization is typically required to prevent intracellular degradation and maintain detection efficacy.

Recent work integrates nanomaterials with electrochemical transducers to achieve real-time NO sensing in complex media. Hemin-functionalized graphene field-effect transistors provide subnanomolar sensitivity and capture NO released from macrophages and endothelial cells under physiological conditions [106]. Metal–organic frameworks (MOFs) acting as nanozymes (e.g., metalloporphyrinic MOFs) catalyze NO redox and enable electrochemical readouts with defined linear ranges and low detection limits, and MOF–enzyme/AuNP composites further improve signal stability and sensitivity [107,108]. In parallel, flexible organic electrochemical transistor platforms allow continuous and wireless in vivo monitoring of NO, complementing optical probes and offering a path toward implantable diagnostics [109].

To address the challenges of NP stability and specificity in complex biological environments, several strategies have been developed. Encapsulation in polymeric or liposomal carriers and PEGylation help to reduce opsonization and proteolytic degradation, thus enhancing in vivo stability. Targeting ligands such as macrophage-specific peptides or antibodies further improve tissue specificity. Moreover, stimuli-responsive nanocarriers—in particular those activated by pathological microenvironments like oxidative stress or enzyme overexpression—allow for controlled release and enhanced sensor performance. Recent reports have demonstrated successful application of polyethylene glycol (PEG)-modified liposomal NPs for inflammation targeting [110], and microenvironment-responsive systems for atherosclerotic plaque delivery [111], highlighting effective strategies to overcome stability and off-target challenges.

Taken together, recent progress in NO detection and diagnostic imaging has not only deepened our understanding of vascular pathophysiology but also established a foundation for therapeutic applications. In the following sections, we therefore turn from diagnostic approaches to NO-based treatments, providing a continuum of “applications of NO in CHD” that integrates both aspects.

## NO-Based Treatments for Atherosclerosis

### Conventional pharmaceuticals

#### NO availability enhancer

Statins attenuate LDL cholesterol biosynthesis via HMG-CoA inhibition, thereby improving endothelium-dependent vasodilation, suppressing vascular inflammation, reducing oxidative stress, inhibiting platelet aggregation and thrombotic events, attenuating platelet-leukocyte adhesion to the vascular endothelium, stabilizing vulnerable plaques, and promoting neovascularization [112,113]. eNOS mediates these pleiotropic effects, as demonstrated by L-NMMA (eNOS inhibitor)-induced blockade of statin benefits [114,115] and abolished responses in eNOS-deficient murine models [116]. Atorvastatin disrupts caveolin-1 (Cav-1)/eNOS interactions, down-regulating Cav-1 expression in endothelial cells and augmenting NO bioavailability [117]. Simvastatin preserves endothelial NO bioactivity by preventing eNOS down-regulation and maintaining its functional coupling [118]. Additionally, statins enhance eNOS expression through post-transcriptional stabilization of eNOS mRNA [119].

Angiotensin-converting enzyme inhibitors (ACEIs) mitigate angiotensin II type 1 receptor (AT1-R) activation, thereby ameliorating endothelial dysfunction and retarding early atherogenesis [120,121]. AT1-R mediates vasoconstriction, extracellular matrix remodeling, platelet aggregation, monocyte adhesion/activation, and inflammatory cytokine release—critical steps in atherosclerotic progression. Angiotensin II promotes atherogenic pathways by up-regulating endothelial oxidized LDL (ox-LDL) receptors, augmenting ox-LDL uptake, and enhancing ROS production [122,123]. Additionally, endothelial angiotensin II drives ROS-dependent NO catabolism [124] and induces eNOS uncoupling via monocyte-mediated S-glutathionylation, reducing NO bioactivity [125]. The PERSPECTIVE trial evaluated perindopril effects on coronary atherosclerosis using angiography and intravascular ultrasound. Post hoc analysis of 118 patients revealed noncalcified plaque regression with perindopril, demonstrating its dual role in vascular functional improvement and structural remodeling [126].

BH_4_ serves as an essential cofactor for NOS, vital for maintaining eNOS functional coupling. Suboptimal BH_4_ levels in cardiovascular cells shift eNOS activity toward superoxide anion production [127]. BH_4_ supplementation improves endothelium-dependent vasodilation in hypercholesterolemic patients [128]. In atherosclerosis, endothelial and macrophage-derived BH_4_ coordinates NOS functional coupling and redox signaling [129]. Douglas et al. crossed Gch1^fl^/^fl^ Tie2cre mice with ApoE^−/−^ mice, demonstrating that 6-week high-fat feeding induced BH_4_ depletion, exacerbated atherosclerotic burden, and increased plaque macrophage content. BH_4_ deficiency in pro-inflammatory macrophages promoted foam cell formation, redox imbalance, and augmented ROS [130]. These findings highlight BH_4_’s role in integrating NOS function and redox homeostasis in atherosclerotic pathogenesis.

L-Arg reverses endothelial dysfunction in hyperlipidemia [131–133], augments coronary artery dilation/stenoses, and reduces post-angioplasty NIH [134], indicating therapeutic potential in atherosclerosis. Cao et al. overexpressed PCSK9 in Sirt3^f/f^ and endothelial-specific Sirt3^EC-KO^ mice via adenovirus, fed high-cholesterol diet for 12 weeks. Sirt3^EC-KO^ mice exhibited accelerated atherosclerosis with macrophage infiltration, inflammation, and reduced L-Arg bioavailability. L-Arg supplementation mitigated plaque formation and inflammation, confirming the protective Sit3–L-Arg axis [135].

Moreover, clinical modalities targeting the eNOS–NO pathway exhibit notable limitations and inconsistencies. Supplementation with BH₄ is hindered by rapid oxidation in circulation, severely reducing its bioavailability and limiting long-term efficacy. L-Arg shows variable effectiveness due to poor correlation between plasma levels and cellular NO production, especially in disease states where cofactor imbalance persists [136]. While statins generally enhance eNOS expression and NO availability through phosphoinositide 3-kinase (PI3K)/protein kinase B (Akt) activation and increased BH₄ regeneration, their anti-inflammatory and endothelial effects display heterogeneity depending on dose, patient cohort, and context [137,138]. ACEIs consistently improve vascular function by reducing oxidative stress and promoting NO signaling, but their impact varies with genetic background and RAS axis dynamics, potentially leading to divergent clinical outcomes [139]. These complexities underscore the necessity for integrating controlled NO delivery systems and personalized therapeutic strategies to achieve reliable endothelial protection.

#### NO donors

NO donor delivery systems have been intensively investigated to optimize NO bioavailability [140,141]. Four principal classes of NO donors include N-diazeniumdiolates (NONOates), RSNOs, nitroaromatics, and metal–nitrosyl complexes. Among these, NONOates represent the most extensively characterized exogenous NO donors [142]. RSNOs, typified by S-nitroso-L-glutathione (GSNO) and S-nitroso-L-cysteine (CysNO), serve as endogenous NO carriers analogous to physiological NO transporters in vivo [112,143]. However, clinical translation of small-molecule NO donors remains challenged by limitations including low payload capacity, uncontrolled burst release, nonspecific biodistribution, and inherent instability to thermal/photochemical degradation [144]. These drawbacks have spurred development of targeted delivery platforms designed to achieve site-specific NO release with precise dosage control. Recent efforts have increasingly focused on improving the stability and controlled release of NO donors through nanomaterial engineering. For example, Wang et al. [145] reviewed advanced NO nanomedicines that integrate chemical conjugation or physical embedding with multifunctional carriers to enhance half-life and minimize systemic toxicity, thereby offering translational potential in cardiovascular therapy. Similarly, Bai et al. [146] reported porous molybdenum nitride nanospheres functioning as efficient, carrier-free NO donors with improved release profiles. These developments underscore how contemporary NO donor systems are being optimized to overcome the inherent instability of traditional small molecules.

Nitro-vasodilators, such as organic nitrates (glyceryl trinitrate [GTN], isosorbide mononitrate [ISMN], and isosorbide dinitrate [ISDN]), remain the primary therapeutic class for atherosclerotic management [147]. Their vasorelaxant effects are mediated through multiple NO-dependent signaling pathways, including RhoA/Rho kinase (ROCK) modulation [148]. GTN, clinically utilized since the 1870s [130], undergoes mitochondrial enzyme-mediated release of 1 molar equivalent of NO. Sublingual administration provides rapid symptomatic relief in acute angina pectoris through transient vasodilation. ISMN and ISDN exhibit delayed onset but prolonged action profiles, making them suitable for chronic CHD prophylaxis [149]. A major limitation of nitrate therapy is the development of tachyphylaxis during continuous administration, characterized by rebound CHD exacerbation upon drug cessation [150]. Proposed mechanisms include impaired enzymatic activation of nitrate prodrugs and desensitization of the sGC signaling axis [151,152].

### Biomaterials

Substantial progress has been achieved in the development of NO delivery systems, leveraging advanced materials science to achieve spatiotemporally controlled NO release at pathological sites. Cutting-edge fabrication techniques, including nanotechnology, electrospinning, and surface modification strategies, enable the incorporation of NO donor prodrugs into diverse biomaterial platforms such as polymeric coatings, NPs, and hydrogels, thereby facilitating sustained, tunable NO bioavailability at target tissues [153,154]. These NO-releasing biomaterials recapitulate endothelial cell function by promoting angiogenesis and maintaining vascular patency [155]. The conjugation of NO donors to biomaterial matrices can be broadly classified into covalent immobilization [156] and physical encapsulation [157–160].

PCI with stent implantation remains the gold-standard treatment for CHD. While drug-eluting stents (DES) incorporating antiproliferative agents or biodegradable polymers have demonstrated efficacy in inhibiting NIH [161–165], their clinical utility is constrained by delayed endothelialization and thrombotic risks arising from nonspecific antiproliferative effects on vascular endothelium [166,167]. Restenosis, defined as luminal narrowing >50% within 6 months of PCI, affects 20% to 50% of patients, driven by complex pathophysiological processes: endothelial denudation triggers platelet activation and aggregation, followed by SMC migration/proliferation mediated by platelet-derived growth factor signaling [168–170].

Pharmacological modulation of platelet behavior has emerged as a critical strategy to mitigate thrombotic complications [171]. Recent advancements in CHD management involve engineering NO-releasing biomaterial coatings (hydrogels, polymers, and metallic nanocomposites) on stent surfaces to locally deliver NO, thereby reducing platelet adhesion/aggregation and restenosis formation (Table 2).

**Table 2.** NO-releasing/generating vascular devices for the treatment of atherosclerosis. NO flux values are reported as mol·cm^−2^·min^−1^ unless otherwise specified. Quantitative effects are extracted directly from the cited studies.

NO origin	Biomaterials utilization	NO flux (mol·cm^−2^·min^−1^)	Model	Outcomes
RSNO (GSNO) catalytic	SeCA–alginate/gelatin hydrogel coating on stent	6.2 × 10^−10^ avg; 2.6 × 10^−9^ peak	In vitro (HUVEC, HUASMC, platelets); in vivo rabbit iliac, pig coronary	SMC migration ↓83%, viability ↓27%; platelet adhesion ≈ 0; NIH thickness ↓32%, stenosis ↓38%; full endothelialization in 1 wk [184]
RSNO (GSNO, SNAP) catalytic	PEI–PEG@GO coating on 3D-printed PLA stent	0.7 × 10^−10^ (GSNO); 1.1 × 10^−10^ (SNAP)	N/A	Conversion: 62% (GSNO), 91% (SNAP); PLA degradation delayed (61% vs. 72.5%) [188]
RSNO (GSNO+GSH) catalytic	HA@DOTA–Cu coating on 316L SS stent	4.0 × 10^−10^ avg; 6.1 × 10^−10^ peak	In vitro (HUVEC, HUASMC, platelets); ex vivo rabbit AV shunt; in vivo rabbit iliac stent	Platelet occlusion ↓87%→12%; SMC migration ↓56%; EC proliferation ↑1.6×; NIH area ↓37% [189]
RSNO (SNAP, GSNO+GSH) catalytic	SeDPA–PPAam coating on 316L SS stent	~3.0 × 10^−10^ steady; 5.6 × 10^−10^ avg	In vitro (HUVEC, HUASMC, platelets); in vivo rabbit iliac stent (28 d)	Platelet adhesion <2%; EC adhesion ↑~100%; NIH stenosis ↓35%→20% [190]
RSNO (GSNO, SNAP) catalytic	SeCA–TiO_2_ (PDA linker) coating on stent	>1.0 × 10^−10^	In vitro (HUASMC, platelets); in vivo dog femoral artery stent (2 mo)	Platelet activation ↓; SMC adhesion ↓; full endothelialization; reduced intimal hyperplasia [191]
RSNO (SNAP, GSNO) catalytic	SeCA–Dopa mussel-inspired coating on 316L SS stent	0.5–2.2 × 10^−10^, stable >60 d	In vitro (HUVEC, HUASMC, platelets); ex vivo rabbit AV shunt; in vivo rabbit iliac stent (1–3 mo)	Platelet adhesion ↓; SMC proliferation ↓; EC adhesion ↑; NIH area and stenosis ↓40%–50% [192]
RSNO (GSNO+GSH) catalytic	Cu^2+^–GA/SeCA coating on 316L SS stent	1–14 × 10^−10^, stable ≥60 d	In vitro (HUVEC, HUASMC, platelets); ex vivo rabbit AV shunt; in vivo rabbit iliac stent (12 wk)	Platelet adhesion ↓; SMC proliferation ↓; EC adhesion ↑; thrombus weight ↓10–20×; blood flow 84% vs. 42%; rabbit ISR ↓44%→31% [193]
RSNO (SNAP+GSH) catalytic	Cu^2+^-PDA coating on 316L SS stent	0.87 × 10^−10^ (Griess); 4.09 × 10^−10^ (NOA)	In vitro (HUVEC, HUASMC, platelets); ex vivo rabbit AV shunt; in vivo rat abdominal aorta (3 wk)	Platelet adhesion ≈ 0; SMC proliferation ↓; EC proliferation ↑; reduced hyperplasia [194]
RSNO (GSNO+GSH) catalytic	DA–Cu^2+^ coating on 316L SS stent	0.4–6.5 × 10^−10^; ~3.8 × 10^−10^ at 30 d	In vitro (HUVEC, HUASMC, platelets); ex vivo rabbit AV shunt; in vivo rabbit iliac stent (1–3 mo)	Ex vivo occlusion ↓90%→0%; thrombus weight ↓38→1 mg; blood flow ↑91% vs. 22%; NIH stenosis ↓42%→13% (3 mo) [195]
RSNO (GSNO+GSH) catalytic	DA–Cu^2+^ coating on 316L SS stent	0–28 × 10^−10^; 44%–61% retained after 15 d	In vitro (HUVEC, HUASMC, platelets); ex vivo rabbit AV shunt	SMC proliferation ↓; EC adhesion ↑; platelet adhesion ↓; ex vivo occlusion ↓91%→0%; thrombus weight ↓ [196]
RSNO (GSNO+GSH) catalytic	CuBTC SURMOF coating (Ti)	5.0 (M10), 9.1 (M20), 13.1 (M30) × 10^−10^, stable ≥30 d	In vitro (HUVEC, HUASMC, platelets, macrophages); ex vivo rabbit AV shunt; in vivo rat abdominal aorta (4 wk)	EC proliferation ↑ (M10); SMC and macrophage proliferation ↓; platelet adhesion ↓; ex vivo occlusion ↓95%→11%; intimal thickness ↓90→40 μm [197]
RSNO (GSNO+GSH) catalytic	Cu^2+^–DA/HD MCAN + BVLD coating on 316L SS stent	0.71–7.47 × 10^−10^, ≥68% retained after 30 d	In vitro (HUVEC, HUASMC, platelets); ex vivo rabbit AV shunt; in vivo rabbit iliac stent (1–12 wk)	Platelet adhesion ↓; SMC proliferation ↓; EC proliferation ↑; ex vivo occlusion ↓100%→5%; NIH area ↓2.4→1.0 mm^2^, stenosis ↓38%→16% [198]
RSNO (GSNO+GSH) catalytic	pDA/Cu^2+^–DA + REDV peptide on 316L SS stent	~6 × 10^−10^; 4.85 ± 0.56 × 10^−10^ with REDV	In vitro (HUVEC, HUASMC, platelets, EC/SMC co-culture); ex vivo rabbit AV shunt; rabbit iliac stent (2 h to 4 wk)	Platelet adhesion ↓; EC adhesion/proliferation ↑; SMC proliferation ↓; full endothelialization (1 wk), reduced restenosis [199]
L-Arg catalytic	iNOS immobilized on 316L SS stent	N/A	In vitro (HUVEC, HUASMC, platelets)	EC adhesion/proliferation ↑; SMC adhesion/proliferation ↓; platelet adhesion almost absent [200]
NONOate donor	Heparin/NONOate NPs on PGMA-coated 316L SS stent	167 μM burst; 385 μM at 10 d	In vitro (HUVECs, whole blood, platelets, RBCs); in vivo rabbit carotid stent (1 mo)	Hemolysis <0.5%; platelet adhesion ↓; EC proliferation ↑; full endothelialization, reduced restenosis [201]
Diazeniumdiolate NO donor	PU graft with NO-releasing polymer (PVC-based)	29.2 × 10^−12^ mol·cm^−2^·s^−1^ (day 1); 5.5 × 10^−12^ mol·cm^−2^·s^−1^ (3 wk)	In vivo sheep AV bridge graft (21 d)	Patency 80% vs. 50% (sham) and 0% (uncoated); thrombus-free area 98% vs. 79% (sham) and 47% (uncoated) [204]
RSNO (GSNO+GSH) catalytic	SePEI/HA + PEG-YIGSR modified PCL graft	41.1 ± 5.1 μM (2 h); 50.6 ± 3.8 μM (4 h)	In vitro (HUVEC, HUASMC, macrophages); in vivo rat abdominal aorta graft (4–8 wk)	Macrophage proliferation ↓; M2 polarization ↑; EC coverage ~50% (4 wk), ~100% (8 wk); reduced intimal hyperplasia [207]
Diazeniumdiolate donor peptide	PU–PEG copolymer with diazeniumdiolate + YIGSR	Burst ~70% (48 h); 86% total release (60 d)	In vitro (BAECs, rat SMCs, platelets, whole blood)	BAEC proliferation ↑; SMC proliferation ↓; platelet adhesion ↓90%–95% [208]
NO gas encapsulated	Echogenic liposomes (phospholipid/cholesterol)	Burst ~50% (10 min); 6 μl/mg lipid, sustained 8 h;	In vitro (rat SMCs ± hemoglobin); in vivo rabbit carotid balloon injury (2 wk)	NO uptake by VSMCs ↑7×; in vivo I/M ratio ↓1.1→0.5; intimal area ↓40%; wall thickness ↓41% [209]

SNAP, S-nitroso-N-acetyl-D-penicillamine; GSNO, S-nitroso-L-glutathione; GSH, glutathione; SeCA, selenocystamine; PDA, polydopamine; DA, dopamine; GA, gallic acid; MCAN, metal–catechol–amine network; HD, hexamethylenediamine; REDV, Arg-Glu-Asp-Val peptide; BVLD, bivalirudin peptide; SURMOF, surface-mounted metal–organic framework; GO, graphene oxide; PEI, polyethyleneimine; PEG, polyethylene glycol; PU, polyurethane; PCL, polycaprolactone; PLA, polylactic acid; PGMA, poly(glycidyl methacrylate); DOTA, 1,4,7,10-tetraazacyclododecane-1,4,7,10-tetraacetic acid; SS, stainless steel; ELIP, echogenic liposomes; HUVEC, human umbilical vein endothelial cell; HUASMC, human umbilical artery smooth muscle cell; BAEC, bovine aortic endothelial cell; RBC, red blood cell; AV shunt, arteriovenous shunt; ISR, in-stent restenosis; I/M ratio, intima/media thickness ratio; N/A, not reported in the original paper

In addition to preclinical and early clinical observations, recent systematic evidence strengthens the translational relevance of NO-based vascular devices. A 2023 meta-analysis of titanium-nitride-oxide (TiNO)-coated stents demonstrated noninferiority to conventional DESs for major adverse cardiovascular events and even a lower incidence of myocardial infarction at 5-year follow-up. These findings provide updated clinical confirmation that NO-related coatings can achieve favorable long-term efficacy and safety in CHD management [172].

#### Hydrogels

NO-releasing hydrogels have emerged as promising platforms for cardiovascular therapeutics and regenerative medicine, offering spatiotemporally controlled bioactive molecule delivery. NapFF-NO, a β-galactosidase-responsive molecular hydrogel, demonstrates sustained NO release over 48 h with a cumulative release efficiency of 35.68% [173]. However, the rapid biodegradation of small-molecule-based systems limits their in vivo applicability. To address this, chitosan-based polymeric hydrogels were engineered to achieve prolonged NO release kinetics (55 h) through covalent conjugation of NO donors to the polysaccharide backbone [174]. Pluronic triblock copolymers [poly(ethylene oxide)-block-poly(propylene oxide)-block-poly(ethylene oxide)] have been extensively explored for NO delivery, with formulations incorporating GSNO or S-nitroso-N-acetylcysteine (SNAC) demonstrating thermo/photoresponsive NO release at pathological sites [175–179]. A notable advancement involved conjugating Pluronic F127 with NONOate prodrugs and branched polyethylenimine (BPEI), which extended the NO release half-life to 1.68 to 2.0 h [180]. To mitigate burst release, Schanuel et al. [181] developed bilayered PVA-SNO/F127 films with gradient NO release profiles, achieving reduced initial peaks followed by exponential decay over 50 min. Multifunctional systems incorporating light-sensitive N-(3-aminopropyl)-3-(trifluoromethyl)-4-nitrobenzenamine or enzyme-responsive O_2_-(2,4-dinitrophenyl)-1-[(4-ethoxycarbonyl)piperazin-1-yl]diazen-1-ium-1,2-diolate further enable stimulus-responsive NO liberation at target sites [182].

In translational research, Taite and West applied NO-releasing PEG copolymer hydrogels to injured carotid arteries in a rat model, achieving near-complete suppression of intimal hyperplasia (90% reduction) at 28 days post-treatment. Histological analysis revealed enhanced endothelial cell migration/proliferation at lesion sites, indicating the hydrogel’s dual capacity to prevent restenosis and promote re-endothelialization [183]. A landmark 2021 study by Chen et al. introduced a NO-eluting (NOE) hydrogel coating for vascular stents (Fig. S2), demonstrating preferential endothelial cell adhesion while inhibiting smooth muscle cell proliferation. In vitro adhesion assays showed 50% fewer human umbilical artery smooth muscle cells (HUASMCs) adhering to NOE hydrogels compared to bare stents, without compromising human umbilical vein endothelial cell (HUVEC) viability. Porcine and rabbit models confirmed accelerated endothelial repair and reduced neointimal formation, establishing NOE hydrogels as a viable strategy for next-generation vascular implants [184].

However, NO-releasing hydrogels face persistent challenges in in vivo applications. Achieving long-term stability, consistent release kinetics, and high biocompatibility remains difficult due to premature degradation of NO donors, burst release, and potential inflammatory responses. Smart hydrogels with stimuli-responsive crosslinking (e.g., pH, light, or enzyme triggers) have shown improved release control and reduced cytotoxicity. Moreover, integrating hydrogels with polymer networks preserves donor stability and localizes NO delivery, enhancing both safety and functional consistency in vivo [185,186].

#### Vascular stents

In recent years, researchers have focused on developing novel NO-releasing stent platforms through surface functionalization strategies, aimed at addressing restenosis and thrombosis. According to platform and material basis, NO-releasing stents can be categorized into 3 main types: polymeric coatings, metallic/catalytic coatings, and nanocomposite/biofunctional coatings.

##### Polymeric coatings

Innovative NO-releasing stent platforms have been engineered through surface functionalization strategies to address restenosis and thrombosis. Do et al. [187] developed PEG-modified poly(lactic-co-glycolic acid) microsphere-incorporated stents that inhibited NIH via cGMP-mediated suppression of VSMC proliferation. An alternative approach involves integrating NO-generating catalysts into stent coatings. Tabish et al. [188] recently reported a bioresorbable 3D-printed stent fabricated from polyethyleneimine–polyethylene glycol–graphene oxide (PEI-PEG@GO) nanocomposites coated onto polylactic acid (PLA) substrates, demonstrating 62% and 91% NO conversion efficiency from 10 μM GSNO and S-nitroso-N-acetylpenicillamine (SNAP) respectively, generating fluxes of 0.7 × 10^−10^ and 1.1 × 10^−10^ (mol·cm^−2^·min^−1^) (Fig. S3). This system promoted endothelial repair while attenuating restenosis through dual mechanisms of NO release and antioxidant activity.

Lyu et al. constructed multifunctional allylamine stents via sequential hydroamidation, immobilizing NO-donating compounds and hyaluronan (HA) to achieve synergistic antithrombotic effects, VSMC inhibition, and endothelial compatibility. In vivo evaluation demonstrated accelerated endothelialization and sustained restenosis prevention over 28 days [189]. Building on this work, the same group developed a catalytic NO-releasing coating using 3,3’-diselenodipropionic acid (SeDPA), which enzymatically converts endogenous RSNOs into bioactive NO. Plasma-polymerized allylamine (PPAam)-immobilized SeDPA coatings exhibited enhanced endothelial coverage and reduced VSMC migration in porcine models [190]. Weng et al. reported titanium dioxide (TiO₂)-based stents functionalized with selenocystamine (SECA), which catalytically decomposed RSNOs to NO. In a canine femoral artery model, SECA-modified stents significantly reduced NIH compared to bare metal controls [191].

A 2015 study by Zhilu et al. introduced a “one-pot” copolymerization strategy to deposit SECA-dopamine (DA) coatings on stainless steel (SS) stents. This system enabled continuous NO release (>60 days) through redox reactions with bloodborne RSNOs, achieving dual effects: (a) inhibition of VSMC proliferation/migration and platelet aggregation via cGMP up-regulation, and (b) promotion of endothelial cell adhesion/proliferation. In vivo evaluation in rabbit carotid models demonstrated complete endothelial coverage by day 14 and 78% reduction in neointimal area compared to uncoated stents [192].

##### Metallic/catalytic coatings

In 2018, Zhilu’s group introduced copper (Cu)-phenolic-amine hybrid coatings capable of catalytic NO generation (1 to 14 × 10^−10^ mol·cm^−2^·min^−1^), which significantly enhanced antithrombotic and restenosis resistance of vascular scaffolds in preclinical models [193]. Li et al. [194] reported DA film-Cu^II^ coatings that enzymatically decomposed endogenous RSNOs to NO, promoting endothelialization and reducing NIH in rabbit carotid models. Zhang et al. [195] developed a biomimetic DA-Cu^II^ coating via sequential immersion, achieving sustained NO release (>30 days) through RSNO decomposition while maintaining endothelial cell selectivity. This system demonstrated 82% reduction in SMC proliferation and 4-fold increase in endothelial cell coverage compared to bare stents. Song et al. [196] validated these findings on 316L SS scaffolds, confirming dose-dependent endothelialization enhancement and restenosis suppression.

Zhao et al. [197] engineered a Cu^II^ benzene-1,3,5-tricarboxylate (CuBTC) MOF coating that decomposed GSNO to NO. In arteriovenous shunt models, CuBTC coatings reduced thrombus formation by 74% and attenuated NIH by 63% compared to controls. Building upon this work, a multifunctional Cu^II^-DA/hexamethylenediamine (Cu^II^-DA/HD) network was developed, combining NO generation with secondary bivalirudin (BVLD) immobilization. The resultant coatings achieved sustained NO release (21 days) while providing complementary anticoagulation, reducing platelet adhesion by 89% and restenosis by 76% in porcine coronary models [198].

In 2020, Xiangyang et al. introduced a mussel-inspired “build-up” strategy for sequential Cu-DA network deposition, enabling precise control over Cu^II^ content (0.8 to 2.3 μg·cm^−2^) and catechol/quinone density (1.2 to 3.5 nmol·cm^−2^). These coatings demonstrated tunable NO release (0.3 to 1.8×10^−10^ mol·cm^−2^·min^−1^) via RSNO decomposition, while surface catechol moieties enabled secondary REDV peptide grafting. The resultant NO-REDV hybrid coatings achieved 92% endothelial cell coverage within 7 days and 84% restenosis reduction in rabbit iliac artery models, outperforming clinically approved DES [199].

##### Nanocomposite/biofunctional coatings

Alagem-Shafir et al. [200] chemically immobilized NOS onto the surface of SS substrates. The functionalized stent demonstrated dual bioactivities: it significantly augmented ECs adhesion and proliferation while concurrently suppressing platelet aggregation and adhesion, thereby exerting antithrombotic and anti-restenotic effects in vascular applications. However, the clinical translatability of this technology is constrained by 2 critical limitations: the ultrathin coating thickness of the stent surface and the inherently low loading capacity of NO donors. These factors collectively impede the sustained release of therapeutically relevant NO concentrations required to facilitate complete vascular endothelialization. As an alternative approach, the endogenous generation of NO through RSNOs represents a promising strategy for achieving prolonged NO bioavailability.

Zhu et al. [201] reported a novel strategy for achieving sustained codelivery of heparin and NO via covalent conjugation of heparin/NONOates NPs onto stent surfaces. In vitro characterization revealed that the polyglycidyl methacrylate (PGMA)-Hep/NONOates-coated SS stent released a substantial burst of NO (~167 μM) within the initial 12-h period, followed by a subsequent sustained release phase achieving a cumulative release of 385.2 μM over 10 days. Rabbit carotid artery implantation studies demonstrated that this multifunctional drug-eluting platform exhibited excellent hemocompatibility, significantly promoted endothelial cell regeneration, and maintained effective anticoagulant properties throughout the observation period. Simon-Walker et al. developed heparin-chitosan polyelectrolyte multilayer-coated TiO_2_ nanotubes, chemically modified with NO donors. These constructs released ~40 pmol NO within 20 min, significantly reducing platelet/leukocyte adhesion and activation [202]. These findings highlight the therapeutic potential of combinatorial heparin-NO delivery systems in improving vascular devices’ performance.

Multifunctional NO-releasing stents, particularly metallic and nanocomposite systems, exhibit superior antithrombotic and restenosis resistance through RSNO decomposition, signaling pathway modulation, and combinatorial drug delivery. These advancements provide a foundation for translating next-generation intelligent stents into clinical practice.

#### Vascular implants

NO-releasing polyurethanes (PUs) have emerged as promising materials for vascular grafts in CABG, addressing thrombotic occlusion through platelet adhesion inhibition and endothelialization promotion.

Prosthetic grafts used in CABG remain susceptible to platelet aggregation, leading to reduced blood flow and graft occlusion. To address this, researchers have developed NO-releasing PUs through strategic incorporation of NO donors, aiming to promote endothelialization and mitigate thrombotic and proliferative responses [203–206]. Fleser et al. employed a dialkylhexanediamine diazeniumdiolate-releasing PU matrix to locally modulate platelet behavior at graft sites. Compared to uncoated controls, NO-releasing grafts exhibited negligible thrombus formation, attributed to NO-mediated inhibition of platelet adhesion to the material surface [204].

Tang et al. engineered a bilayered vascular graft using electrospun polycaprolactone (PCL) as the structural backbone, incorporating organoselenium-functionalized polyethyleneimine (SePEI) for in situ NO generation via electrostatic layer-by-layer assembly, and HA conjugated with PEG-modified YIGSR peptides for endothelial cell targeting. In rat carotid artery models, these grafts induced macrophage polarization toward an anti-inflammatory (M2) phenotype, promoted endothelial remodeling, and suppressed nonspecific protein adsorption after 4 to 8 weeks of implantation [207].

Taite et al. [208] integrated YIGSR peptides into NO-releasing PU-PEG copolymers, demonstrating enhanced endothelial cell adhesion and migration. Huang et al. developed echogenic liposomal carriers (ELIPs) encapsulating 1,2-dipalmitoyl-sn-glycero-3-ethylphosphocholine, 1,2-dioleoyl-sn-glycero-3-phosphocholine, and cholesterol, achieving NO loading efficiency of 10 μl/mg via pressurized freezing. Compared to free NO, ELIP-encapsulated NO improved delivery to injured rabbit carotid arteries by 7-fold, resulting in significant attenuation of NIH [209].

These systems demonstrate dual efficacy in reducing thrombotic complications and promoting vascular healing, offering innovative solutions for small-diameter graft challenges. Among them, hydrogel-coated vascular implants appear most promising for cardiovascular use, offering controlled release, biocompatibility, and endothelial healing benefits.

Future optimization of NO-based biomaterials should focus on achieving spatiotemporally precise release profiles that mimic the dynamic signaling of endogenous endothelial NO. Emerging strategies include enzyme- or ROS-responsive platforms that enable “on-demand” NO generation at lesion sites, and multi-phase release systems that recapitulate basal physiological fluxes while providing transient surges under pathological conditions. Importantly, fine-tuning NO flux to physiological ranges (0.5 to 4 × 10^−10^ mol·cm^−2^·min^−1^) can minimize cytotoxicity and mitigate tolerance development. Integration with antioxidant codelivery (e.g., BH_4_ and GSH) and surface-anchored catalytic coatings further ensures localized, sustained activity while avoiding systemic exposure. Such approaches will be instrumental in developing next-generation NO-biomaterials capable of precision vascular therapy.

## NO-Related Nanoparticle Systems

Advanced nanomaterials have been engineered to address cardiovascular pathologies through targeted NO delivery. This section focuses on free NP carriers and nano-assemblies that are not anchored to intravascular devices. Nano-enabled stent/implant coatings have been discussed in the preceding sections. Compared with bulk coatings, NP systems offer unique advantages including high surface-to-volume ratios, tunable surface chemistry, and responsiveness to biological stimuli, which enable precise spatiotemporal control of NO release.

### Silica nanoparticles

Silica nanoparticles (SNPs) have gained substantial traction in biomedical applications due to their tunable size, facile synthesis, and robust biocompatibility [210,211]. NO-functionalized silica nanostructures are typically synthesized via sol-gel methodologies, which can be categorized into pre-synthetic and post-synthetic strategies depending on the functionalization sequence [212–214]. Carpenter et al. [214] employed reverse microemulsion techniques to prepare amine-functionalized SNPs of 3 distinct sizes with comparable amine densities, followed by covalent immobilization of NONOate prodrugs onto the NP surface.

Two core–shell architectures were developed using silica cores and RSNO-modified chitosan shells. In the first route, azetidine-2,4-dione-functionalized SNPs were reacted with chitosan chains, followed by thioglycolic acid (GA-SH) conjugation to yield SNP/CH-SH-I. Alternatively, thiol-functionalized chitosan was pre-grafted onto silica cores to form SNP/CH-SH-II. Subsequent nitrosation generated SNP/CH-SNO-I and SNP/CH-SNO-II, releasing 0.10 and 0.17 mmol·g^−1^ NO, respectively [215].

Hetrick et al. [216,217] utilized a pre-synthetic approach to incorporate diazeniumdiolate moieties into silica matrices, achieving 6-fold higher NO payload (0.3 μmol·mg^−1^) compared to post-synthetically functionalized counterparts. Malone-Povolny and Schoenfisch embedded RSNO-modified mesoporous silica nanoparticles (MSNs) into PU matrices, demonstrating sustained NO release (>30 days) without NP leaching. This composite maintained 90% of its NO payload after 4 days of storage at 0 °C [218]. Although embedded into a PU matrix, this design was still fundamentally NP-driven, as the MSN carriers provided the primary reservoir for NO release.

### Diverse nanoparticulate systems

Kushwaha et al. [219] engineered self-assembling peptide amphiphile (PA) nanofibrous matrices incorporating NO-donating residues and YIGSR peptides, demonstrating dual functionality: NO release promoted EC proliferation while attenuating SMC proliferation and platelet adhesion. Solvent evaporation-based coating of these nanofibers with YIGSR- or polylysine (KKKKK)-conjugated NO-releasing PAs yielded analogous therapeutic effects [220]. Rink et al. developed reconstituted high-density lipoprotein (rHDL) NPs by embedding RSNO-modified phospholipids within the HDL shell via 3H-formaldehyde-mediated reductive methylation. These functionalized rHDL particles retained native HDL properties while delivering bioactive NO, reducing atherosclerotic plaque burden by 42% in ApoE^−/−^ mice fed a high-fat diet [221].

Bahnson et al. [222] designed targeted NO-delivery nanofibers bearing collagen-binding peptides and S-nitrosylated cysteine residues, achieving site-specific NO release in rat carotid balloon injury models. Intravenous administration reduced NIH by 68% compared to controls. Singha et al. [223] fabricated hybrid NO-releasing CarboSil PUs incorporating zinc oxide (ZnO) NPs loaded with SNAP, maintaining sustained NO flux (>0.5 × 10^−10^ mol·cm^−2^·min^−1^) over 72 h. Such hybrid PU systems illustrate how NP incorporation can endow bulk polymers with NO release functionality, while still being conceptually grounded in NP-enabled delivery.

### Micelles

The hydrophilic nature of NO donors poses substantial challenges for their encapsulation within hydrophobic cores, prompting researchers to conjugate these donors to amphiphilic block copolymers for constructing micellar nanostructures that enable efficient delivery [224–226]. Davis et al. employed reversible addition-fragmentation chain transfer polymerization to synthesize a diblock copolymer (Vitamin-POEGMA-b-VDM), onto which GSNO was covalently linked via azlactone functional groups. This conjugation strategy produced monodisperse spherical micelles measuring 50 nm in diameter, capable of sustained NO release with a half-life exceeding 14 days, in contrast to the short half-life (1 to 2 days) observed for free GSNO [225,227]. This observation underscores the efficacy of nanoparticulate encapsulation in extending the NO release kinetics of donor molecules.

Advanced nanosystems have been engineered using multifunctional materials and innovative architectures to achieve spatiotemporally controlled NO liberation, thereby ameliorating thrombotic complications, restenosis, and atherosclerotic progression while minimizing systemic toxicity. A recent comprehensive review also highlights the therapeutic potential of NO-based nanomedicines in CVDs, including myocardial ischemia–reperfusion injury, emphasizing targeted delivery and multimodal strategies [228].

**Fig. 3.**
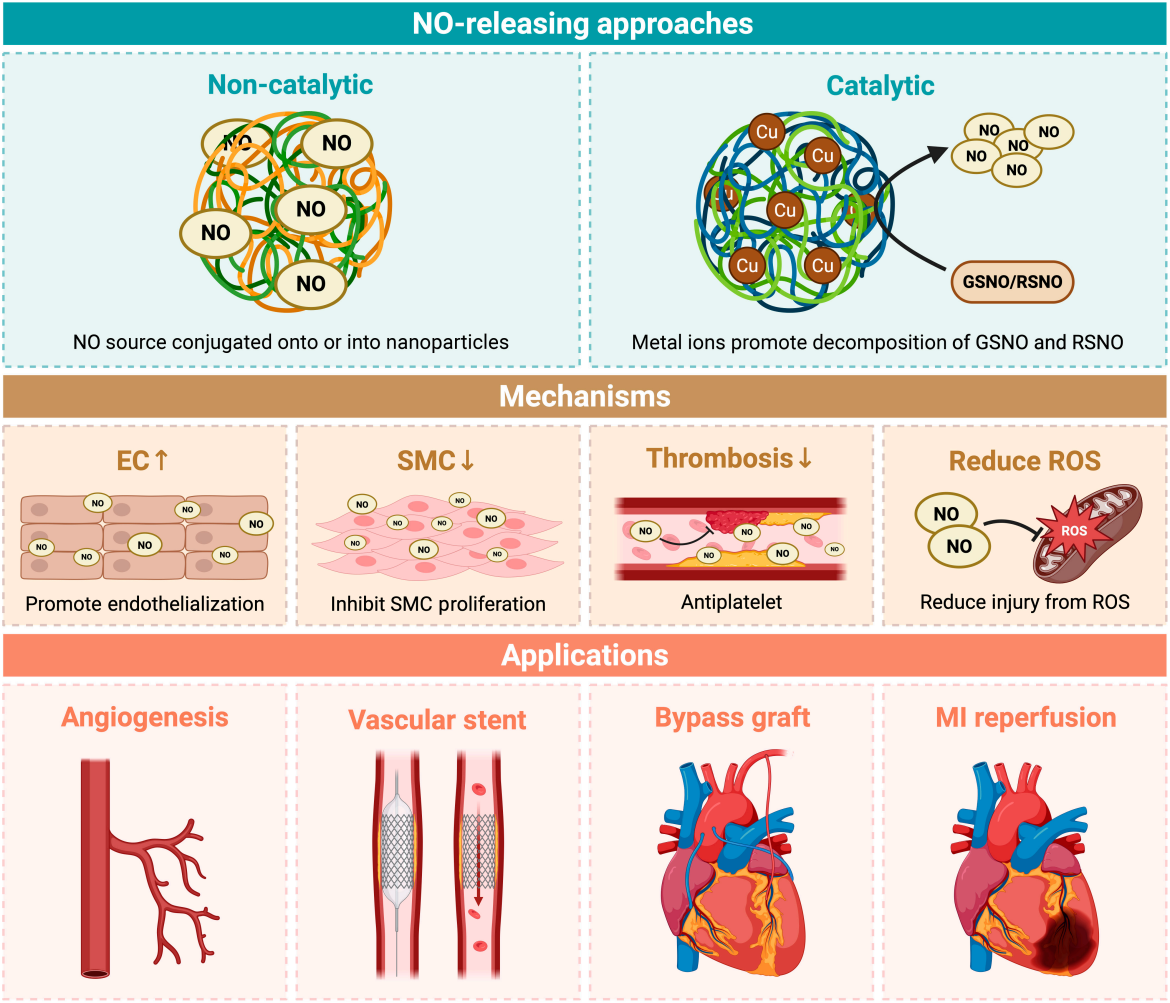
Conceptual illustration of NO-responsive nanocarriers in atherosclerosis. Created in BioRender.

NO-responsive nanomaterials, designed with spatiotemporal NO delivery precision, hold substantial translational potential across cardiovascular and noncardiovascular applications. Compared with polymeric or metallic coatings, NP platforms provide unique advantages, including systemic administration, plaque targeting, and integration with imaging or therapeutic functions. Preclinical studies have demonstrated their ability to attenuate thrombosis, restenosis, and atherosclerotic progression, while applications are also emerging in oncology and wound repair. Representative studies are summarized in Table 3, and Fig. 3 conceptually illustrates how NO-responsive nanocarriers may intervene in atherosclerotic progression. Despite limited mechanistic insights in atherosclerosis, these nanosystems highlight promising avenues for CHD management. Future work should focus on pharmacokinetics, biosafety, and clinical translation pathways to fully realize the therapeutic potential of NO-based nanomedicines.

**Table 3.** Representative NO-related nanoparticle systems for cardiovascular applications

Variation	Biomaterials utilization	NO releasing rate	Outcomes/Applications
Silica@chitosan NPs	RSNO (chitosan shell)	0.10–0.17 mmol SNO/mg; *T*_1/2_ ~10 h, release up to 39 h	Prolonged NO stability; antimicrobial and biomedical carrier applications [215]
Silica NPs (AHAP3 or MAP3)	NONOate (diazeniumdiolate)	AHAP3: 3.8 μmol·mg^−1^, [NO]_m_ 21,700 ppb·mg^−1^, *T*_1/2_ ~18 min; MAP3: 7.6 μmol·mg^−1^, [NO]_m_ 190,000 ppb·mg^−1^, *T*_1/2_ ~6 min	Broad-spectrum anti-biofilm; Low fibroblast toxicity [216]
QA-functionalized silica NPs	NONOate (diazeniumdiolate-modified amines)	~0.27–0.30 μmol·mg^−1^; [NO]_max_ 617–1,388 ppb/mg; *T*_max_ 1.5–4.3 min	Dual action; QA+NO synergistic; low fibroblast toxicity [217]
PU + RSNO-MSNPs	RSNO (MSNs)	Max flux ~1,040 ppb·mg^−1^; *T*_1/2_ ~26 h; duration ~90 h; payload 2.18 μmol·mg^−1^ (up to 3.29 μmol·mg^−1^ with Cu^2+^)	Long-term NO delivery; anti-thrombosis and anti-inflammation [218]
PA nanofibrous matrix	Lysine-derived NONOate (PA-KKKKK)	Burst 48 h, sustained 30 d (~0.32 μmol)	HUVEC proliferation ↑ (67% vs. 51%); AoSMC proliferation ↓ (16% vs. 35%); platelet adhesion ↓150-fold [219]
Electrospun PCL+PA	NONOate (PA-KKKKK)	Burst 48 h, sustained 28 d (~3.8 μmol/disc)	HUVEC proliferation ↑ (70% PCNA^+^); AoSMC proliferation ↓ (compared to control); platelet adhesion ↓ (~90%) [220]
HDL-like NPs (AuNP core + ApoA-I + phospholipid bilayer)	RSNO (DPPNOTE)	~33% release at 4 h, ~80% at 24 h, stable with GSH-triggered release	Targeted delivery via SR-B1 receptor; NF-κB activation in macrophages ↓; AoSMC migration ↓ (~89%); protects against ischemia/reperfusion injury in mouse kidney transplant; ApoE^−^/^−^ mice atherosclerotic plaque↓ (42%) [221]
Collagen-targeted PA nanofibers	RSNO (Cys)	*T*_1/2_ ~49 s (ascorbate/Cu^2+^); ~90% theoretical yield within 24 h	Targeted to carotid injury; VSMC proliferation ↓; NIH ↓up to 7 mo; I/M ratio ↓ (55%); Occlusion ↓ (41% at 2 wk) [222]
CarboSil + SNAP + ZnO NPs	RSNO (SNAP) + ZnO catalyst	>0.5 × 10^−10^ mol·cm^−2^·min^−1^ for 14 d	Synergistic antimicrobial; SNAP leaching ↓ (55%); noncytotoxic to fibroblasts [223]
PEG-b-PNORM-b-PEG micelles	DNP derivative	~100 μM under 410 nm irradiation (73% yield)	Light-triggered NO + DOX release; reversed MDR; anticancer efficacy ↑[224]
Polymeric NPs (RAFT)	RSNO (GSNO conjugated)	*T*_1/2_ >14 d; ~90% release within 24 h (5 mM ascorbate)	Enhanced NO donor stability; efficient intracellular NO release; synergistic with cisplatin; nontoxic to fibroblasts [225]
Vitamin A-decorated NPs	RSNO (GSNO conjugated)	~80% release within 24 h	Targeted HSC uptake; fibrosis markers ↓ (40%–60%); HSC proliferation and migration ↓; portal pressure ↓ (~25%) [226]
Star polymer NPs	NONOate (spermine)	~355 nM·h^−1^ for 70 h	Inhibits *P. aeruginosa* biofilm formation ↓ (90%–95%); dispersal to planktonic state ↓; nontoxic at effective doses [227]

NP, nanoparticle; RSNO, S-nitrosothiol; QA, quaternary ammonium; PU, polyurethane; MSN, mesoporous silica nanoparticle; PA, peptide amphiphile; PCL, polycaprolactone; NONOate, diazeniumdiolate; HUVEC, human umbilical vein endothelial cell; AoSMC, aortic smooth muscle cell; DPPNOTE, S-nitrosylated phospholipid; SR-B1, scavenger receptor class B type 1; Cys, cysteine; NIH, neointimal hyperplasia; SNAP, S-nitroso-N-acetylpenicillamine; Ti, titanium; DNP, N,N′-dinitroso-p-phenylenediamine; DOX, doxorubicin; RAFT, reversible addition–fragmentation chain transfer; GSNO, S-nitroso-L-glutathione; Asc, ascorbate; Vit A, vitamin A; HSC, hepatic stellate cell; MDR, multidrug resistance; LOQ, limit of quantification; BAC, benzalkonium chloride

## Conclusions and Prospects

NO remains a central regulator of vascular homeostasis, exerting vasodilatory, anti-inflammatory, and antithrombotic functions that are highly relevant in CHD. Over the past decade, biomaterial-based strategies—including catalytic coatings, NO-donor–loaded hydrogels, and engineered NPs—have substantially advanced, demonstrating the ability to restore NO signaling in preclinical models and offering versatile diagnostic and therapeutic opportunities.

These achievements illustrate the translational promise of NO-centered interventions. However, critical challenges persist, including the limited half-life of NO, the need for spatiotemporal control of release, potential cytotoxicity at supraphysiological levels, and uncertainties regarding long-term biosafety. Addressing these challenges requires multidisciplinary efforts to integrate materials science, vascular biology, and regulatory standards.

Looking forward, future directions should prioritize (a) the development of scalable, reproducible fabrication processes, (b) integration of multifunctional platforms that combine NO delivery with anti-inflammatory or antioxidant cues, (c) advanced imaging and sensing approaches to monitor local NO flux in vivo, and (d) rigorous evaluation in clinically relevant large-animal models. These strategies will accelerate the path toward clinical translation.

Importantly, beyond these general conclusions, specific translational bottlenecks—such as scalability, therapeutic safety windows, and recent clinical evidence—warrant dedicated discussion.

## Translational Challenges and Clinical Outlook

### Scalability and reproducibility

Despite encouraging preclinical efficacy, scalability and batch-to-batch reproducibility remain central hurdles for NO-releasing biomaterials. Variations in donor loading or catalytic site density (e.g., selenocystamine content in catalytic hydrogels) can shift NO flux and alter biological outcomes, underscoring the need for in-process controls and release testing that directly quantify NO production. Synthetic hydrogel platforms are generally more amenable to scale-up with minimal batch variation, but flux uniformity must still be demonstrated across lots. Implementing chemiluminescence-based NO rate measurements as a quality attribute, alongside conventional physical/chemical assays, provides a robust bridge from bench formulations to clinical-grade materials [184,229].

### Therapeutic window and biosafety

The physiological endothelium-mimicking NO flux is commonly referenced as 0.5 to 4 × 10^−10^ mol·cm^−2^·min^−1^. Insufficient delivery fails to restore endothelial function, whereas excessive exposure risks cytotoxicity and platelet dysfunction, partly via nitrosative stress pathways. Accordingly, clinical translation should target tunable, endothelium-range flux with safeguards against burst release, and explicitly report dose metrics in flux units to facilitate cross-study comparison [230,231].

### Clinical landscape

While NOE coronary stents remain confined to large-animal studies (e.g., hydrogel-coated stents improving re-endothelialization and reducing restenosis in rabbits and pigs) [184], other NO platforms have progressed into human trials. The ProNOx1 randomized clinical trial (NCT01982565) evaluated an NO-generating hydrogel dressing (EDX110) in diabetic foot ulcers, reporting significant improvements in wound closure and favorable safety outcomes [232]. As a related clinical benchmark for hemocompatible surfaces, titanium–nitride–oxide (TiNO)-coated stents (non-NOE) demonstrated noninferiority to everolimus-eluting stents with 5-year follow-up in acute coronary syndrome (ACS) patients in TIDES-ACS (*n* = 1,491), highlighting the value of drug-free bioactive surfaces while NOE stent technologies mature [233]. These examples provide valuable translational lessons regarding safety evaluation, durability, and regulatory acceptance.

### Regulatory alignment

For device-based approaches, integration with ISO 25539-2:2020 (vascular stent evaluation) and Food and Drug Administration-recognized consensus standards is necessary. Defining NO flux as a reportable metric—alongside mechanical integrity and hemocompatibility—would harmonize performance benchmarks across laboratories and manufacturers [234].

### Outlook

Moving forward, successful translation of NO-based biomaterials will depend on (a) reproducible, scalable fabrication, (b) precise control of NO flux within therapeutic ranges, (c) incorporation of standardized assays and regulatory-aligned evaluation, and (d) clinical trial designs that capture both safety and vascular functional endpoints. With these steps, NO-releasing biomaterials may progress from experimental promise to clinical impact in CHD.

## Data Availability

This review did not generate new data. All data and information included are from previously published sources cited in the manuscript.
