# Supplementary material for: Nitric Oxide in Biomaterial-Based Therapies for Coronary Heart Disease: Mechanistic Insights, Current Advances, and Translational Prospects
Source: Biomater Res. 2025 Oct 9;29:0267. doi: 10.34133/bmr.0267 (PMC12509227; doi:10.34133/bmr.0267)
Supplement: Supplementary 1 — Figs. S1 to S3 [file bmr.0267.f1.zip › Supplemental Materials.docx]

**Supplemental Materials**


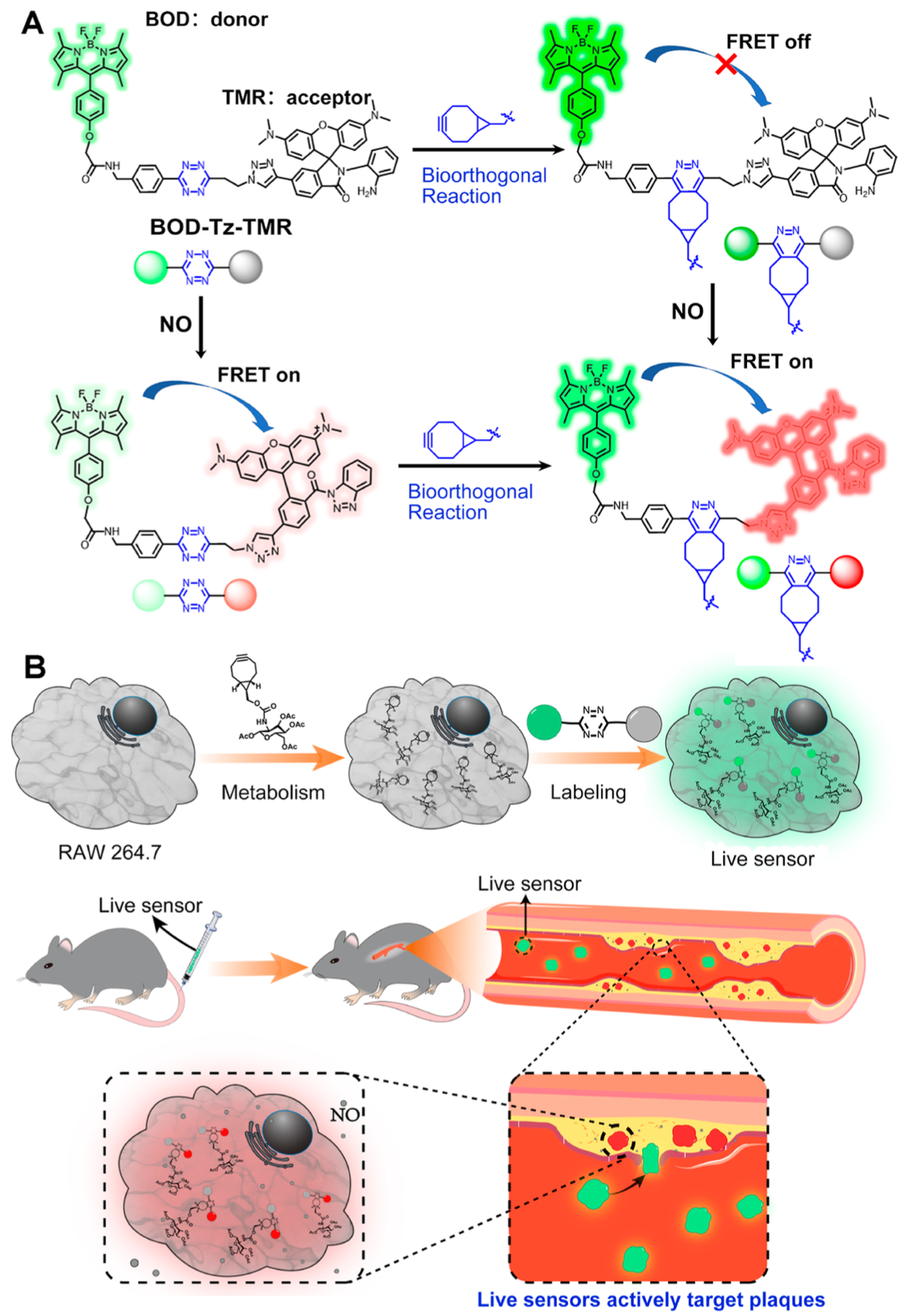


**Figure S1. (A)** Molecular Mechanism of the Probe BOD-Tz-TMR for Bio-orthogonal Reaction and Response to NO. **(B)** Engineering and In Vivo Sensing Mechanism of the Live Sensor. (Reprinted from ref.^96^. Copyright (2023) American Chemical Society).


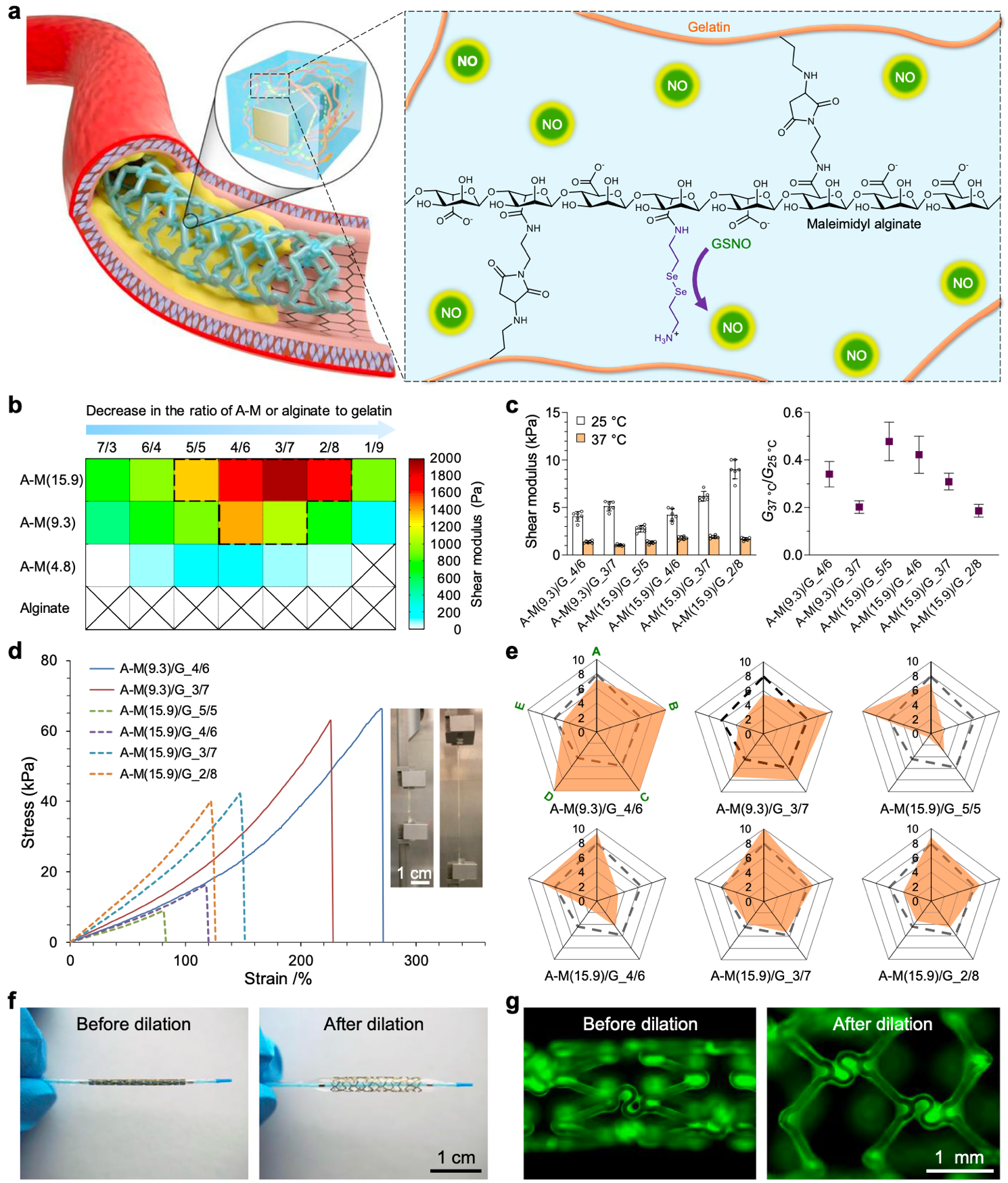


**Figure S2. Development of a tough NOE hydrogel coating for vascular stent. (a)** Schematic for the design of our NOE hydrogel. **(b)** Shear moduli (at 37 °C) of the hybrid hydrogels formulated with maleimidyl alginate (A–M) of varying degrees of modification (DM) and gelatin (G) at different mass ratios. **(c)** Comparison in shear modulus among the selected hydrogels at 25 °C and 37 °C (mean ± SD, n = 6 independent samples). **(d)** Tensile testing of them at ambient temperature. The insets exhibit the photographs of A–M(9.3)/G_4/6 hydrogel prior to and during extension. **(e)** Radar charts showing their scores in shear modulus (at 37 °C) (A), fracture strength (B), fracture strain (C), toughness (D), and capacity for further modification (E). Dashed frameworks represent the average values. **(f)** Photographs of a vascular stent coated with A–M(9.3)/G_4/6 hydrogel before and after balloon dilation in PBS at 37 °C. **(g)** Fluorescence images of it before and after balloon dilation. The hydrogel coating was labeled with fluorescein isocyanate. (Reprinted from ref.^184^. Copyright (2021) Springer Nature).


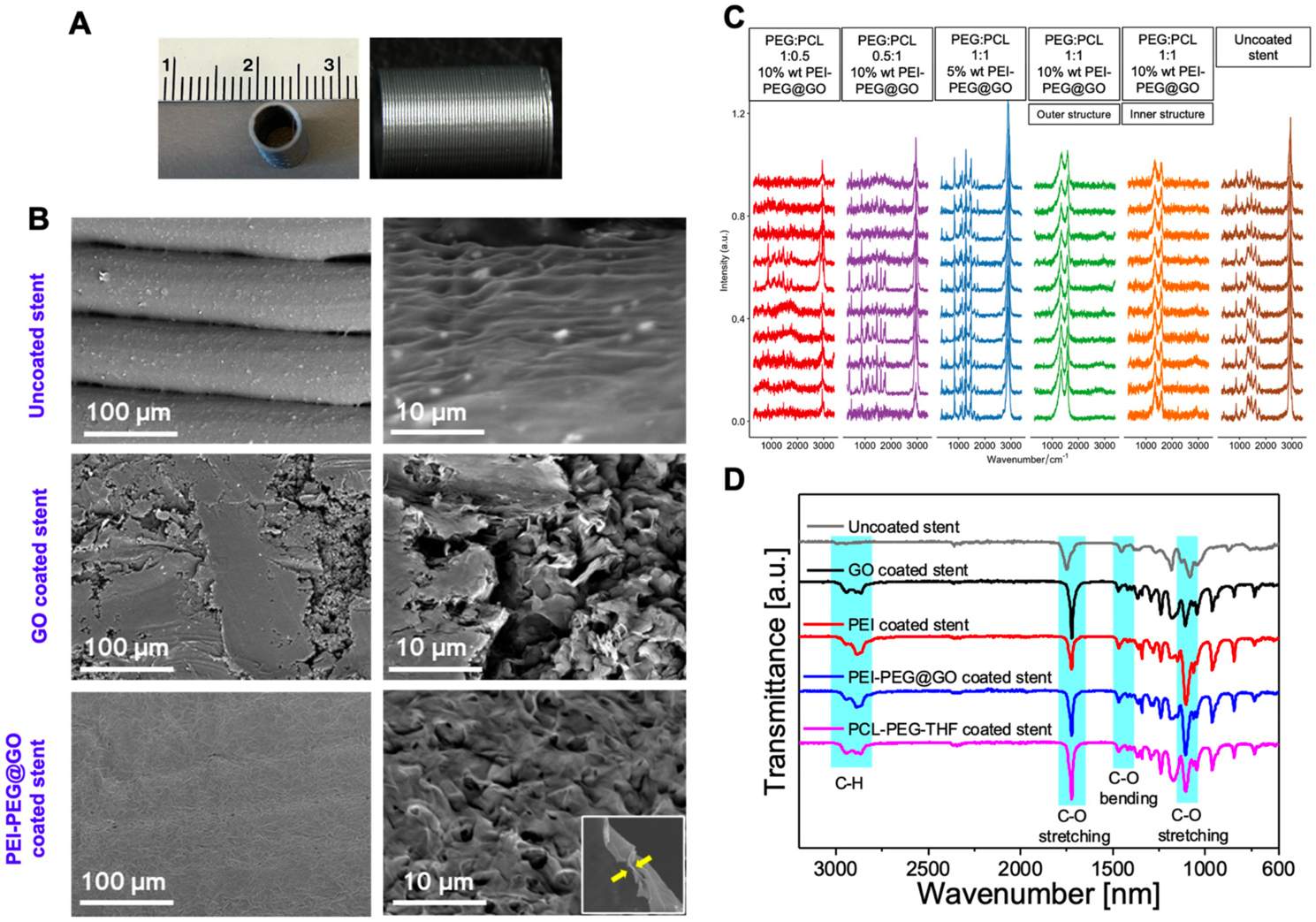


**Figure S3. Characterization of the coating of PEI-PEG@GO on the stent material (PLA). (A)** Photographs of 3D-printed stents. **(B)** SEM micrographs of coated and uncoated stents at different magnifications. The inset figure in PEI-PEG@GO coated stent shows the average thickness of coating which is 0.2 μm. **(C)** Raman spectra of uncoated and coated stents (with PEI-PEG@GO) using different combinations of coating solutions and PEI-PEG@GO. Three random fields of view were chosen with ~18 random points mapping per field view along with ~54 points per stent. The optimized coating solution (PEG:PCL 1:1 and 10 wt. % PEI-PEG@GO) was prepared after screening different ratios of PEG and PCL in THF with PEI-PEG@GO. The weight percentage of PEG, PCL, and PEI-PEG@GO in the coating solution is 47.5%, 47.5%, and 5%, respectively. Each spectrum in this panel represents characterization at a spatially distinct sampling point. **(D)** FTIR spectra of uncoated and coated stents under different conditions. The characteristic FTIR bands are shown along with region names. (Reprinted from ref.^188^. Copyright (2024) AIP Publishing LLC).
