# Supplementary figures and images for: Nitric Oxide in Biomaterial-Based Therapies for Coronary Heart Disease: Mechanistic Insights, Current Advances, and Translational Prospects
Source: Biomater Res. 2025 Oct 9;29:0267. doi: 10.34133/bmr.0267 (PMC12509227; doi:10.34133/bmr.0267)

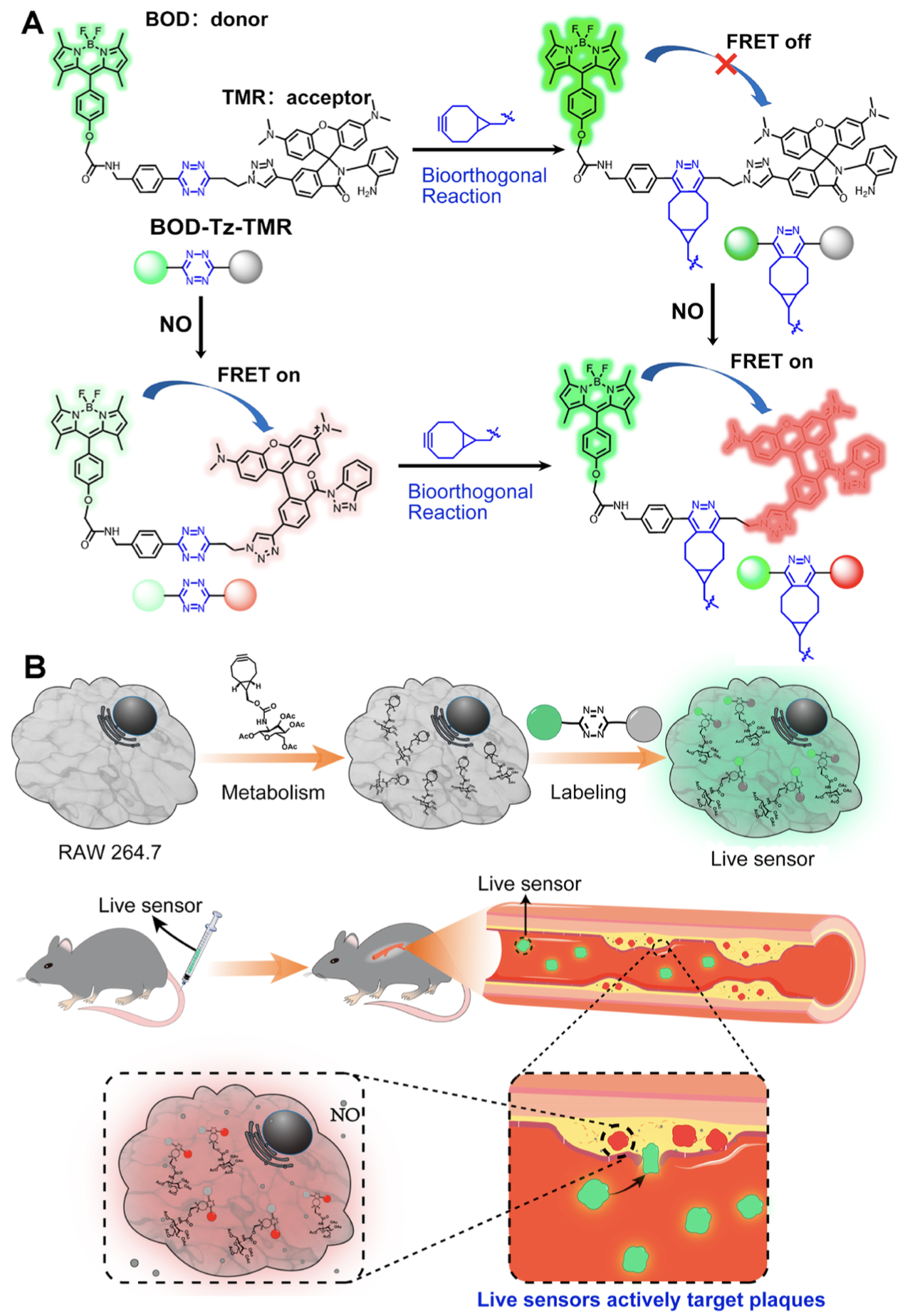

Supplement: Supplementary 1 — Figs. S1 to S3 [file bmr.0267.f1.zip › Figure S1.png]

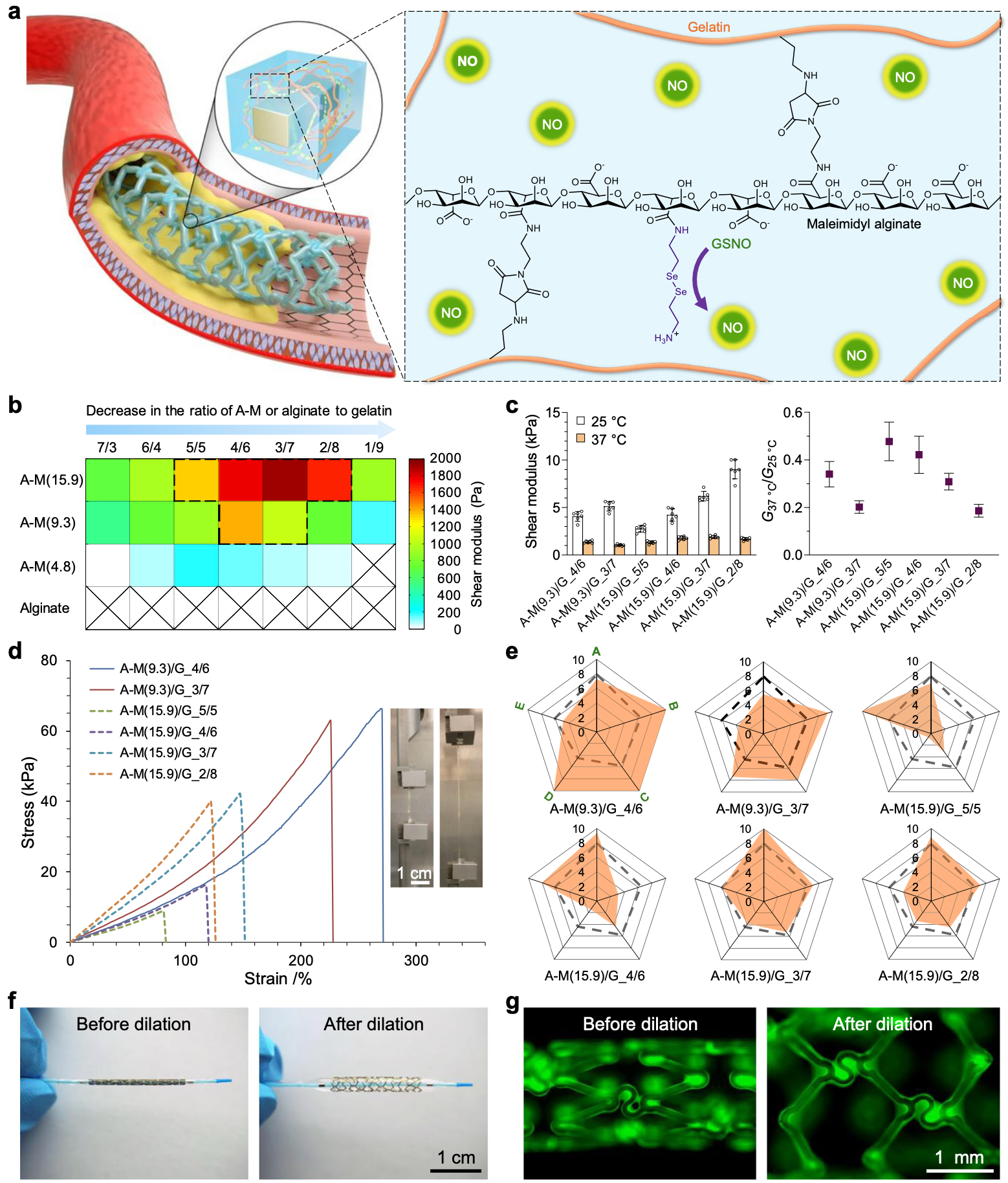

Supplement: Supplementary 1 — Figs. S1 to S3 [file bmr.0267.f1.zip › Figure S2.png]

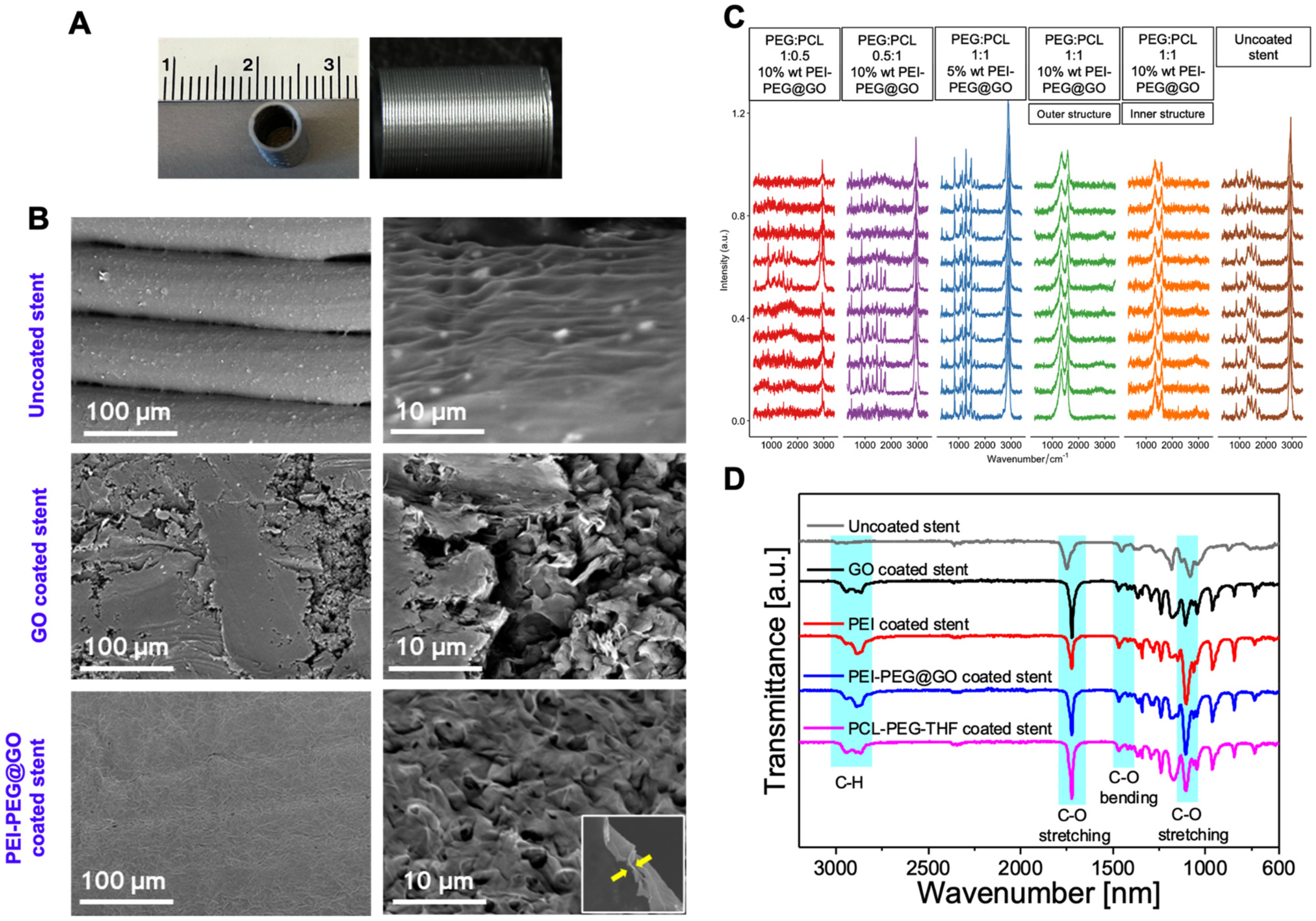

Supplement: Supplementary 1 — Figs. S1 to S3 [file bmr.0267.f1.zip › Figure S3.png]
